# Insulin Inhibits Nrf2 Gene Expression via Heterogeneous Nuclear Ribonucleoprotein F/K in Diabetic Mice

**DOI:** 10.1210/en.2016-1576

**Published:** 2017-01-23

**Authors:** Anindya Ghosh, Shaaban Abdo, Shuiling Zhao, Chin-Han Wu, Yixuan Shi, Chao-Sheng Lo, Isabelle Chenier, Thierry Alquier, Janos G. Filep, Julie R. Ingelfinger, Shao-Ling Zhang, John S. D. Chan

**Affiliations:** 1 Department of Medicine, Université de Montréal and Centre de recherche du Centre hospitalier de l’Université de Montréal (CRCHUM), Montreal, Quebec H2X 0A9, Canada; 2 Department of Pathology and Cell Biology, Université de Montréal and Centre de recherche, Hôpital Maisonneuve-Rosemont, Montreal, Quebec H1T 2M4, Canada; 3 Pediatric Nephrology Unit, Massachusetts General Hospital, Harvard Medical School, Boston, Massachusetts 02114-3117

## Abstract

Oxidative stress induces endogenous antioxidants via nuclear factor erythroid 2–related factor 2 (Nrf2), potentially preventing tissue injury. We investigated whether insulin affects renal Nrf2 expression in type 1 diabetes (T1D) and studied its underlying mechanism. Insulin normalized hyperglycemia, hypertension, oxidative stress, and renal injury; inhibited renal *Nrf2* and angiotensinogen (*Agt*) gene expression; and upregulated heterogeneous nuclear ribonucleoprotein F and K (*hnRNP F* and *hnRNP K*) expression in Akita mice with T1D. In immortalized rat renal proximal tubular cells, insulin suppressed *Nrf2* and *Agt* but stimulated *hnRNP F* and *hnRNP K* gene transcription in high glucose via p44/42 mitogen-activated protein kinase signaling. Transfection with small interfering RNAs of *p44/42 MAPK, hnRNP F*, or *hnRNP K* blocked insulin inhibition of *Nrf2* gene transcription. Insulin curbed *Nrf2* promoter activity via a specific DNA-responsive element that binds hnRNP F/K, and hnRNP F/K overexpression curtailed *Nrf2* promoter activity. In hyperinsulinemic-euglycemic mice, renal *Nrf2* and *Agt* expression was downregulated, whereas *hnRNP F/K* expression was upregulated. Thus, the beneficial actions of insulin in diabetic nephropathy appear to be mediated, in part, by suppressing renal *Nrf2* and *Agt* gene transcription and preventing Nrf2 stimulation of *Agt* expression via hnRNP F/K. These findings identify hnRNP F/K and Nrf2 as potential therapeutic targets in diabetes.

Under physiological conditions, oxidative stress triggers upregulation of endogenous antioxidants via nuclear factor erythroid 2–related factor 2 (Nrf2), which may prevent tissue injury by the induction of genes encoding various antioxidant and phase 2-detoxifying enzymes (1–3). Preclinical studies have postulated a renoprotective role for Nrf2 activation in diabetes (4–6). Clinical trials with bardoxolone methyl (an Nrf2 activator that activates Nrf2 signaling and also inhibits nuclear factor-*κ*B and STAT signaling in human cell lines) (7, 8), however, have yielded conflicting results. The phase 2 Trial to Determine the Effects of Bardoxolone Methyl on eGFR in Patients With Type 2 Diabetes and Chronic Kidney Disease study targeted renoprotective actions of bardoxolone methyl in patients with type 2 diabetes with stage 3b or 4 chronic kidney disease (9). In contrast, the phase 3 Bardoxolone Methyl Evaluation in Patients With Chronic Kidney Disease and Type 2 Diabetes (BEACON) trial in type 2 diabetes patients with stage 4 chronic kidney disease was discontinued after 9 months owing to insufficient improvement in renal function and unchanged risk of end-stage renal disease (10). Bardoxolone methyl actually increased the risk of heart failure and cardiovascular death in the BEACON trial (10). The reasons for these disparate outcomes remain unknown.

We reported previously that catalase (Cat) overexpression in renal proximal tubular cells (RPTCs) prevents hypertension and nephropathy, attenuates renal angiotensinogen (*Agt*) and *Nrf2* gene expression, and blocks Nrf2 stimulation of *Agt* gene transcription, in type 1 diabetes (T1D) Akita Cat-transgenic mice (11–13). Our data suggested that chronic Nrf2 activation by hyperglycemia might aggravate renal dysfunction via enhanced intrarenal renin-angiotensin system (RAS) in diabetes.

Beyond its hypoglycemic effect, insulin has been shown to regulate the expression of transcription factor genes and genes involved in inflammation and insulin signaling (14, 15). We previously established that insulin inhibits high glucose (HG) and reactive oxygen species (ROS) stimulation of renal *Agt* expression via 2 nuclear proteins, heterogeneous nuclear ribonucleoprotein F and K (hnRNP F and hnRNP K), which bind to a putative insulin-responsive element (*IRE*) in the rat *Agt* gene promoter (16–21). We further established that hnRNP F normalizes systemic hypertension via suppression of renal Agt production in transgenic mice specifically overexpressing hnRNP F in their RPTCs (22). Recently, we showed that hnRNP F and hnRNP K mediate, at least in part, insulin suppression of renal *Agt* gene expression (23).

Here we investigated whether insulin could inhibit *Nrf2* gene transcription, avert Nrf2-stimulation of *Agt* gene expression via hnRNP F/K, and, subsequently, prevent systemic hypertension and renal injury in T1D mice.

## Materials and Methods

### Chemicals and constructs

d-glucose, d-mannitol, human insulin, PD98059 [a p44/42 mitogen-activated protein kinase (p44/42 MAPK) inhibitor], wortmannin and Ly-294,002 (specific inhibitors of phosphatidylinositol 3-kinase), and oltipraz (an Nrf2 activator) were purchased from Sigma-Aldrich Canada Ltd. (Oakville, ON, Canada). U0126 (a p44/42 MAPK inhibitor) was obtained from Cell Signaling Technology (New England Biolabs Ltd., Whitby, ON, Canada). Dulbecco’s modified Eagle medium (DMEM, 5 mmol/L d-glucose, catalog no. 12320) and penicillin/streptomycin and fetal bovine serum were procured from Invitrogen, Inc. (Burlington, ON, Canada). Insulin implants (Linßit, with a release rate of approximately 0.1 unit/implant/day for >30 days) were sourced from Linshin (Scarborough, ON, Canada). pGL4.20 [Luc/Puro] vector containing luciferase reporter came from Promega Corporation (Sunnyvale, CA). The pGL4.20 construct, containing the rat *Agt* gene promoter *N*-1495 to N+18 or the rat *Nrf2* gene promoter *N*-1960 to N+111, has been described previously (11, 24). The *hnRNP F* gene promoter *N*-1,500 to N+99 and the *hnRNP K* gene promoter *N*-1,516 to N+16 were cloned from rat genomic DNA by conventional polymerase chain reaction (PCR) with specific primers (Table 1), confirmed by DNA sequencing, and then inserted into pGL4.20 vector via Kpn I and Hind III restriction sites. Rabbit polyclonal antibodies specific to rat hnRNP F and polyclonal antibodies against rat Agt were generated in our laboratory (J.S.D.C.) (20, 25). The other antibodies used are listed in Table 2. Scrambled Silencer Negative Control #1 and *p44/42 MAPK*, *Nrf2*, *hnRNP F*, and *hnRNP K* small interfering RNAs (siRNAs) were provided by Ambion, Inc. (Austin, TX). Restriction and modifying enzymes were supplied by Invitrogen, Inc., and New England Biolabs. Oligonucleotides were synthesized by Integrated DNA Technologies (Coralville, IA). QuickChange II Site-Directed Mutagenesis Kit and LightShift Chemiluminescent electrophoretic mobility shift assay (EMSA) Kit were procured from Agilent Technologies (Santa Clara, CA) and Thermo Scientific (Life Technologies Inc., Burlington, ON, Canada), respectively. Primer biotin-labeling kit was purchased from Integrated DNA Technologies.

**Table 1.** **Primer Sequences for RT-qPCR, Subcloning, and EMSA**

**Gene/Species**	**Forward/Reverse Primer Sequences**	**Reference Sequence**
Angiotensinogen (mouse/rat)	F: 5′-CCACGCTCTCTGGATTTATC-3′	NM_007428.3
	R: 5′-ACAGACACCGAGATGCTGTT-3′	
HO-1 (mouse/rat)	F: 5′-CACCAAGTTCAAACAGCTCT-3′	NM_010442.2
	R: 5′-CAGGAAACTGAGTGTGAGGA-3′	
hnRNP F (mouse/rat)	F: 5′-AATTGTGCCAAACGGGATCA-3′	NM_133834.2
	R: 5′-GCACCAGACCTCATCCTATCCA-3′	
hnRNP K (mouse/rat)	F: 5′- CAGCTCCCGCTCGAATCTG-3′	NM_001301341.1
	R: 5′- ACCCTATCAGGTTTTCCTCCAA-3′	
KEAP1 (mouse/rat)	F: 5′-CATCCACCCTAAGGTCATGGA-3′	NM_016679.4
	R: 5′-GACAGGTTGAAGAACTCCTCC-3′	
Nrf2 (mouse/rat)	F: 5′-CGCCGCCTCACCTCTGCTGCCAGTAG-3′	NM_010902.3
	R: 5′-AGCTCATAATCCTTCTGTCG-3′	
Nox1 (mouse/rat)	F: 5′-GGTCACTCCCTTTGCTTCCA-3′	NM_172203.2
	R: 5′- GGCAAAGGCACCTGTCTCTCT-3′	
Nox2 (mouse/rat)	F: 5′-CCCTTTGGTACAGCCAGTGAAGAT-3′	NM_007807.5
	R: 5′- CAATCCCGGCTCCCACTAACATCA-3′	
Nox4 (mouse/rat)	F: 5′-TGGCCAACGAAGGGGTTAAA-3′	NM_015760.4
	R: 5′-GATGAGGCTGCAGTTGAGGT-3′	
*β*-Actin (mouse/rat)	F: 5′-ACGATTTCCCTCTCAGCTT-3′	NM_031144.3
	R: 5′-TACAATGAGCTGCGTGTGGC-3′	
hnRNP F gene promoter (rat)	F: 5′-AAAGGTACCTTTTTAAAGTCTTAAGCATTTG-3′	NC_005103.4
	R: 5′-AAAAAGCTTCAGGGGAAACGCTTTTCG-3′	
hnRNP K gene promoter (rat)	F: 5′-AAAGGTACCGGAGGCAACGGCGGACTCGC-3′	NC_005116.4
	R: 5′-AAAAAGCTTACCAATTCACCATTGGTTTCGG-3′	
Rat Nrf2 promoter	F: 5′-TAATTAGGTACCCTTGCCTCTTGCCCTAGCC-3′	−150
	F: 5′-TAATTAGGTACCCCCGAACCACGAGAGGAGG-3′	−400
	F: 5′-TAATTAGGTACCTTCGGCAAACAGCTGCTAATC-3′	−537
	F: 5′-TAATTAGGTACCAGCGTGGACTCATCCATCTC-3′	−820
	R: 5′-AAAAAACTCGAGTGCTGGGACTGTAGTCCTGGC-3′	+111
Rat Nrf2 promoter- hnRNP F/K-RE (*N*-607/-592)	F: 5′-CGATAGCAGCGCAGGTGTGTTTGCTC-3′	Site-directed mutagenesis primers
	R: 5′-GAGCAAACACACCTGCGCTGCTATCG-3′	
Rat Nrf2 promoter- hnRNP F/K-RE (*N*-463/-444)	F: 5′-CAAGGCCTCCTGCTACTTCAGCCCAC-3′	Site-directed mutagenesis primers
	R: 5′-GTGGGCTGAAGTAGCAGGAGGCCTTG-3′	
Rat Nrf2 promoter hnRNP F/K-RE (*N*-463/-444)	F: 5′-CTCGCGCCCCGCCCCCGCGGGAC-3′	Biotinylated probe for EMSA
	R: 5′-GTCCCGCGGGGGCGGGGCGCGAG-3′	
Rat Nrf2 promoter hnRNP F/K-RE (*N*-463/-444) WT	F: 5′-CTCGCGCCCCGCCCCCGCGGGAC-3′	Competitor
	R: 5′-GTCCCGCGGGGGCGGGGCGCGAG-3′	
hnRNP F/K-RE (M1)	F: 5′-CTCGCG**AAAA**GCCCCCGCGGGAC-3′	Competitor
	R: 5′-GTCCCGCGGGGGC**TTTT**CGCGAG-3′	
hnRNP F/K-RE (M2)	F: 5′-CTCGCGCCC**AAA**CCCCGCGGGAC-3′	Competitor
	R: 5′-GTCCCGCGGGG**TTT**GGGCGCGAG-3′	
hnRNP F/K-RE (M3)	F: 5′-CTCGCGCCCCG**AAAA**CGCGGGAC-3′	Competitor
	R: 5′-GTCCCGCG**TTTT**CGGGGCGCGAG-3′	
hnRNP F/K-RE (M4)	F: 5′-CTCGCG**AAAA**G**AAAA**CGCGGGAC-3′	Competitor
	R: 5′-GTCCCGCG**TTTT**C**TTTT**CGCGAG-3′	

Boldface letters indicate the nucleotides replacing the nucleotides in WT hnRNP F/K-RE.

Abbreviations: HO-1, heme oxygenase-1; Keap1, Kelch-like ECH-associated protein 1; RE, responsive element; RT-qPCR, real-time quantitative polymerase chain reaction.

**Table 2.** **Antibodies Used in This Study**

**Protein Target**	**Name of Antibody**	**Manufacturer, Catalog, and/or Name of Individual Providing the Antibody**	**Species Raised in; Mono or Polyclonal**	**Dilution for WB and or IHC**	**RRID**
Agt	Angiotensinogen antibody	Specifically recognizing Agt were generated in our laboratory	Rabbit; polyclonal	WB; 1:2000	AB_2631321
				IHC; 1:200	
Cat	Catalase	Sigma-Aldrich	Rabbit; polyclonal	WB; 1:1000	AB_259018
				IHC;1:200	
HO-1	HO-1 antibody	Enzo Life Sciences (SPA-895(D))	Rabbit; polyclonal	WB; 1:2000	AB_2248405
				IHC; 1:200	
hnRNP F	hnRNP F antibody	Specifically recognizing (CTARRYIGIVKQAGLER) were generated in our laboratory	Rabbit; polyclonal	WB; 1:10,000	AB_2631323
				IHC; 1:200	
hnRNP K	hnRNP K (H-300)	Santa Cruz Biotechnology (sc-25373)	Rabbit; polyclonal	WB; 1:1000	AB_2120388
				IHC; 1:100	
hnRNP K	Anti–hnRNP K antibody (3C2)	Abcam (ab39975)	Mouse; monoclonal-chip grade	—	AB_732981
Keap1	Anti-Keap1	Abcam (ab66620)	Rabbit; polyclonal	WB; 1:1500	AB_1141055
				IHC; 1:200	
Nrf2	Anti-Nrf2	Abcam (ab31163)	Rabbit; polyclonal	WB; 1:1000	AB_881705
				IHC; 1:200	
*β*-Actin	*β*-Actin clone AC-15	Sigma-Aldrich (A5441)	Mouse; monoclonal	WB; 1:20,000	AB_476744
pERK1/2	Phospho-p44/42 MAPK (Thr202/ Tyr204) (E10)	Cell Signaling (#9106)	Mouse; monoclonal	WB; 1:1000	AB_331768
ERK1/2	p44/42 MAPK	Cell Signaling (#9102)	Rabbit; polyclonal	WB; 1:2000	AB_330744
p-Nrf2	Nrf2 (S40)	Bioss (bs-2013R)	Rabbit; polyclonal	WB; 1:1000	AB_10855428

Abbreviations: IHC, immunohistochemistry; RRID, Research Resource Identifier.

### Physiological studies

Adult male wild-type (WT) and heterozygous Akita mice with mutated *insulin2* gene (C57BL/6-Ins2^Akita^/J) were purchased from Jackson Laboratories (Bar Harbor, ME).

Male Akita mice (age 10 weeks) were divided into 2 groups with and without insulin implants at week 12 until week 16 (23). Non-Akita littermates served as controls. All animals had access to standard mouse chow and water *ad libitum*. Animal care and procedures were approved by the Centre de recherche du Centre hospitalier de l’Université de Montréal Animal Care Committee and followed the Principles of Laboratory Animal Care [National Institutes of Health (NIH) publication no. 85-23, revised 1985: http://grants1.nih.gov/grants/olaw/references/phspol.htm].

Blood glucose levels and systolic blood pressure (SBP) were measured with an Accu-Chek Performa System (Roche Diagnostics Laval, Quebec, Canada) and BP-2000 tail-cuff pressure monitor (Visitech Systems, Apex, NC), respectively (11–13, 22, 23, 26). The mice were housed individually in metabolic cages 24 hours before euthanasia. Blood was collected by cardiac puncture before euthanization and centrifuged for serum. Urine was sampled and assayed for albumin/creatinine ratio (ACR) by enzyme-linked immunosorbent assay with Albuwell and Creatinine Companion (Exocell, Inc., Philadelphia, PA) (11–13, 22, 23, 26).

Glomerular filtration rate (GFR) was estimated with fluorescein isothiocyanate inulin (11, 22, 23, 26). Kidneys were removed immediately after GFR measurement, decapsulated, and weighed before Percoll gradient isolation of renal proximal tubules (RPTs) (11, 22, 23, 26). Aliquots of freshly isolated RPTs from individual mice were immediately processed for total RNA and protein isolation.

Separate hyperinsulinemic-euglycemic clamp experiments were performed on conscious male C57Bl/6 mice (age 12 to 14 weeks) after a 4-hour food restriction (27).

### Serum and urinary Agt and angiotensin II

Serum and urinary Agt and angiotensin II (Ang II) levels were quantified by enzyme-linked immunosorbent assay (Immuno-Biological Laboratories, Inc., Minneapolis, MN) (11, 13, 22, 23, 26).

### Morphologic studies

Kidney sections (3 to 4 μm thick, 4 to 5 sections per kidney, 5 to 6 kidneys per group) were stained with standard periodic acid Schiff or Masson’s trichrome or processed for immunohistochemistry (ABC Staining, Santa Cruz Biotechnology, Santa Cruz, CA) (11–13, 22, 23, 26). Tubular luminal areas, mean glomerular tuft, and RPTC volumes were assessed on periodic acid Schiff–stained sections (11–13, 22, 23, 26). Immunostained images were quantified by NIH ImageJ software (http://rsb.info.nih.gov/ij/).

ROS generation as an index of oxidative stress was assessed by dihydroethidium (DHE; Sigma-Aldrich Canada Ltd.) staining of frozen kidney sections (11–13, 22, 23, 26) and by lucigenin in freshly-isolated RPTs (11–13, 17–19, 22, 23, 26). The results were confirmed by standard Cat and nicotinamide adenine dinucleotide phosphate (NADPH) oxidase activity assays (22, 23, 26, 28).

### Effect of Insulin on gene expression in immortalized renal proximal tubular cells

Rat immortalized renal proximal tubular cells (IRPTCs) (29) (passages 12 to 18) were studied. Plasmids pGL4.20-*Agt*, pGL4.20-*Nrf2*, pGL4.20-*hnRNP F*, and pGL4.20-*hnRNP K,* respectively, containing *Agt, Nrf2, hnRNP F*, and *hnRNP K* gene promoters, were transfected into IRPTCs. Stable transformants were selected in the presence of 0.6 mg/L of puromycin (11).

To study the effects of insulin, stable transformants (75% to 85% confluence) were synchronized overnight in serum-free DMEM containing 5 mmol/L d-glucose, then incubated in normal glucose (NG, 5 mmol/L d-glucose plus 20 mmol/L d-mannitol) or HG (25 mmol/L d-glucose) DMEM containing 1% depleted fetal bovine serum and insulin (10^−7^ mol/L) for up to 24 hours ± p44/42 MAPK inhibitors (PD98059 or U0126), phosphatidylinositol 3-kinase inhibitors (Ly-294, 002 or wortmannin), or the Nrf2 activator oltipraz. The cells were then harvested, and promoter activity was measured by luciferase assay (11, 23, 30). IRPTCs stably transfected with pGL4.20 served as controls.

In additional studies, stable IRPTC transformants were transfected with scrambled siRNA, *p44/42 MAPK, Nrf2, hnRNP F*, or *hnRNP K* siRNAs (11, 23, 30), and their effects on gene promoter activity, messenger RNA (mRNA), and protein expression were analyzed after 24 hours of culture.

### Real-time quantitative polymerase chain reaction assays and Western blotting

*Cat, Agt, hnRNP F, hnRNP K, Nrf2,* heme oxygenase-1 (*HO-1),* Kelch-like ECH-associated protein 1 (*Keap1), Nox1, Nox2, Nox4*, and *β*-*actin* mRNA levels in RPTs and IRPTCs were quantified by real-time quantitative PCR (RT-qPCR) with specific primers (Table 1).

Western blotting (WB) was undertaken (11–13, 22, 23, 26, 28, 30). The relative densities of Cat, Agt, hnRNP F, hnRNP K, Nrf2, HO-1, Keap1, and *β*-actin bands were quantified by computerized laser densitometry (ImageQuant software, version 5.1, Molecular Dynamics, Sunnyvale, CA).

### Statistical analysis

Values were expressed as mean ± standard error of the mean (SEM). Data were analyzed using 1- or 2-way analysis of variance, as appropriate, followed by a Bonferroni multiple comparison testing. *P* < 0.05 values were considered statistically significant.

## Results

### Physiological studies

Table 3 reports the results of physiological measurements in non-Akita WT, Akita, and Akita mice treated with insulin at the age of 16 weeks. Insulin normalized blood glucose, SBP, kidney weight/tibia length (KW/TL) and heart weight/TL ratios, ACR, GFR, urinary Agt, and Ang II levels in Akita mice compared with untreated Akita controls. No changes in serum Agt levels were detected among the different groups.

**Table 3.** **Physiological Measurements**

	WT	Akita	Akita + Insulin
Blood glucose (mmol/L)	7.46 ± 0.667	31.6 ± 0.76^*a*^	14.62 ± 3.57^*b*^
Systolic blood pressure (mm Hg)	109.17 ± 1.5	133.2 ± 4.86^*a*^	114.3 ± 4.16^*c*^
Body weight (g)	30.7 ± 0.73	22.41 ± 0.45^*a*^	24.35 ± 0.42^*a*^
Kidney weight (mg)	324 ± 11	520 ± 27^*a*^	467 ± 10^*a*^^,^^*d*^
Heart weight (mg)	140 ± 10	160 ± 10	150 ± 10
Tibia length (mm)	18.5 ± 0.15	16.3 ± 0.12^*a*^	17.3 ± 0.10^*c*^^,^^*e*^
Kidney/tibia length (mg/mm)	17.51± 0.8	31.91 ± 1.36^*a*^	26.99 ± 0.02^*a*^^,^^*c*^
Heart/tibia length (mg/mm)	7.6 ± 0.10	9.8 ± 0.31^*e*^	8.6 ± 0.10^*d*^^,^^*f*^
ACR (µg/µmol)	1.12 ± 0.17	5.64 ± 0.32^*a*^	1.96 ± 0.10^*b*^
GFR/body weight (µL/min^−1^g^−1^)	6.65 ± 0.12	16.3 ± 0.37^*a*^	7.79 ± 0.48^*b*^
Urinary Agt/creatinine ratio (ng/mg)	29.44 ± 4.3	289.75 ± 61.2^*a*^	167.6 ± 21.1^*a*^^,^^*c*^
UrinaryAng II/creatinine ratio (ng/mg)	1.40 ± 0.42	23.64 ± 12.04^*e*^	5.10 ± 5.01^*a*^^,^^*b*^
Serum Agt (ng/mL)	5221 ± 43.4	4609 ± 78.73	4114.13 ± 95.01
Glomerular tuft volume (×10^3^ μm^3^)	141.2 ± 4.52	182.03 ± 6.3^*a*^	135.7 ± 6.61^*b*^
RPTC volume (×10^3^ μm^3^)	6.9 ± 0.66	9.93 ± 0.27^*a*^	7.81 ± 0.37^*c*^^,^^*f*^
Tubular luminar area (μm^2^)	44.7 ± 5.01	71.75 ± 4.02^*a*^	54.54 ± 6.03^*c*^

^a^ *P* < 0.005 vs WT.

^b^ *P* < 0.005 vs Akita.

^c^ *P* < 0.01 vs Akita.

^d^ *P* < 0.05 vs WT.

^e^ *P* < 0.01 vs WT.

f *P* < 0.05 vs Akita.

### Histological studies

Consistent with earlier observations (11, 13, 22, 23, 26, 28, 30), Akita mice developed renal damage, including proximal tubule cell atrophy, tubule lumen dilation, accumulation of cell debris [Supplemental Fig. 1(a)], and increased extracellular matrix proteins in the glomeruli and tubules [Supplemental Fig. 1(b)]. Glomerular tufts, RPTC volume, and renal tubule lumen areas were augmented significantly in Akita mice compared with WT controls. Insulin treatment normalized these changes (Table 3).

**Figure 1.**
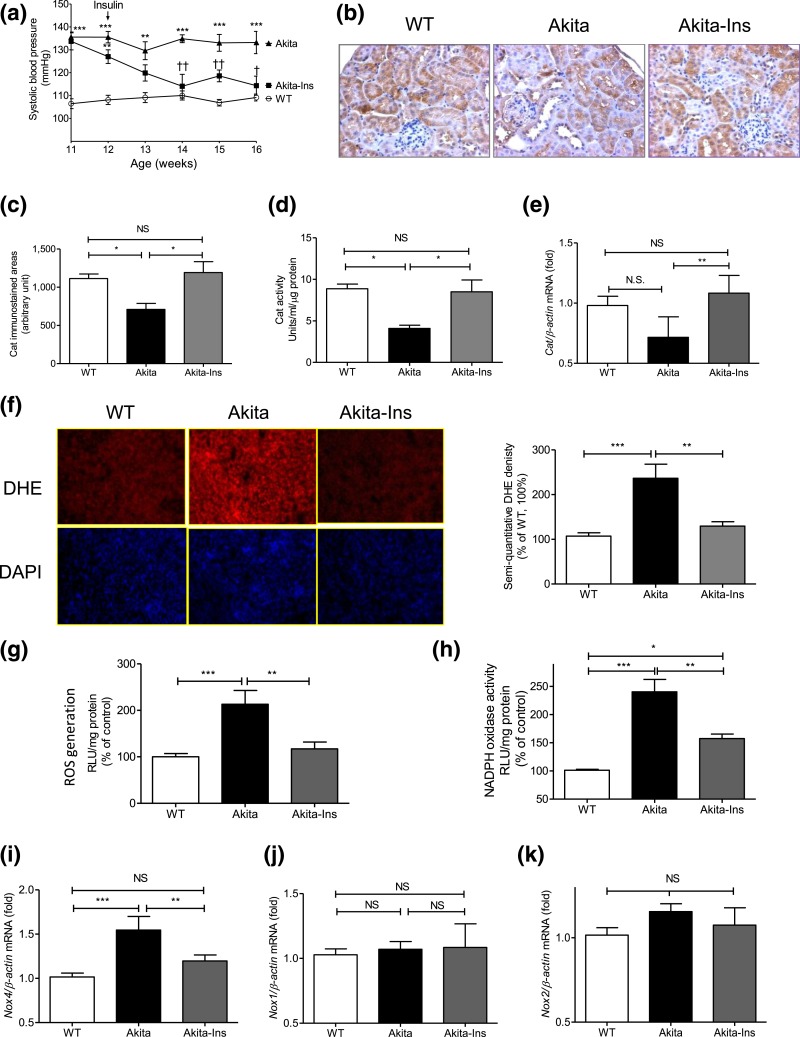
Insulin prevents systemic hypertension and renal oxidative stress in Akita mice. (a) Longitudinal changes in mean SBP (measured 2 to 3 times per mouse per week in the morning without fasting). Baseline SBP was recorded daily over 5 days before initiation of measurements. (b) Cat immunostaining, (c) semiquantitation of Cat-immunostained areas, (d) Cat activity, (e) *Cat* mRNA level, (f) DHE (red) staining (left panel) and semiquantitation of DHE fluorescence (right panel), (g) ROS generation by lucigenin assay, (h) NADPH oxidase activity, (i) *Nox4*, (j) *Nox1*, and (k) *Nox2* mRNA expression in freshly isolated RPTs from WT controls, Akita mice, and Akita mice + insulin (Ins) implants. Values are mean ± SEM, n = 8 per group. *P < 0.05; **P < 0.01, and ***P < 0.005, WT vs Akita. ^††^*P* < 0.01, Akita vs Akita-Ins. WT controls (open bars); Akita (solid bars), and Akita mice + Ins (gray bars). DAPI, 4′, 6-diamidino-2-phenylindole; NS, not significant; RLU, relative luciferase unit.

Average SBP was 20 to 25 mm Hg higher in Akita mice at age 11 weeks than in WT mice (*P* < 0.005) and remained significantly elevated for the study’s duration [Fig. 1(a); Table 3]. Insulin treatment completely normalized SBP in Akita mice.

Cat immunostaining [Fig. 1(b)] and semiquantitation of Cat-immunostained areas [Fig. 1(c)], Cat activity [Fig. 1(d)], but not *Cat* mRNA expression [Fig. 1(e)], were significantly lower in RPTs from Akita vs WT mice. Insulin treatment reversed these changes in Akita mice. In contrast, Akita mice exhibited significantly greater DHE staining [Fig. 1(f)], ROS levels [Fig. 1(g)], NADPH oxidase activity [Fig. 1(h)], and *Nox4* mRNA expression [Fig. 1(i)] than WT controls. Insulin normalized these changes. No differences in *Nox1* and *Nox2* mRNA expression were detected [Fig. 1(j) and 1(k)].

### Renal Agt, HO-1, Nrf2, Keap1, and hnRNP F/K expression

**Figure 2.**
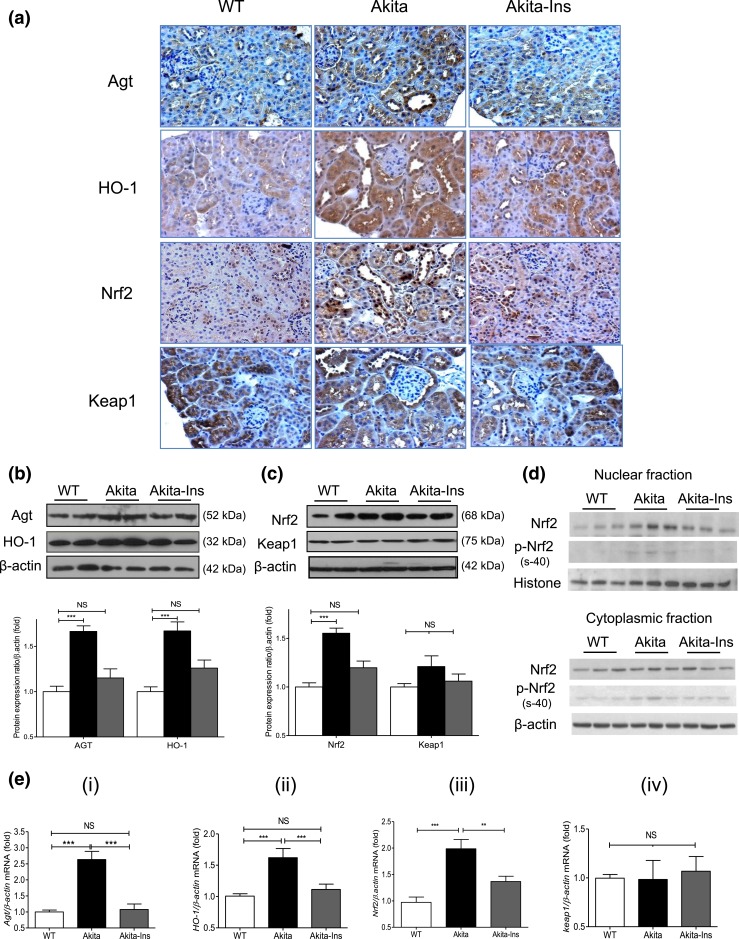
Renal Agt, HO-1, Nrf2, and Keap1 expression in Akita mice. (a) Agt, HO-1, Nrf2, and Keap1 immunostaining (magnification ×200). (b) WB of Agt and HO-1 in total lysates. (c) WB of Nrf2 and Keap1 in total lysates. (d) WB of Nrf2 and *p*-Nrf2 (s-40) in nuclear and cytoplasmic fractions of RPTs. (e) (i–iv) RT-qPCR of *Agt*, *HO-1, Nrf2*, and *Keap1* mRNA expression in RPTs of WT controls, Akita, and Akita mice + insulin (Ins). Values are mean ± SEM, n = 8 per group. **P < 0.01; ***P < 0.005; WT controls (open bars); Akita (solid bars), and Akita mice + Ins (gray bars). NS, not significant.

Agt, HO-1, and Nrf2 immunostaining increased in RPTCs of Akita mice compared with WT controls. Treatment with insulin normalized these changes [Fig. 2(a)]. Keap1 immunostaining did not differ between groups [Fig. 2(a)]. WB of Agt and HO-1 [Fig. 2(b)], Nrf2 and Keap1 [Fig. 2(c)], and RT-qPCR of *Agt*, *HO-1, Nrf2*, and *Keap1* mRNA expression [Fig. 2(e), i–iv] from isolated RPTs confirmed these findings. Furthermore, insulin treatment decreased nuclear Nrf2 and phosphorylated (p)-Nrf2 (s-40) expression without significantly affecting cytosolic Nrf2 and *p*-Nrf2 expression in RPTs of Akita mice [Fig. 2(d)]. Consistent with previous observations (23), immunostaining of hnRNP F/K and WB of hnRNP F/K showed decreases in Akita compared with WT mice, with normalization by insulin [Supplemental Fig. 1(c), i and ii, respectively].

### Effect of insulin on *Agt*, *hnRNP F/K*, and *Nrf2* gene expression in IRPTCs

**Figure 3.**
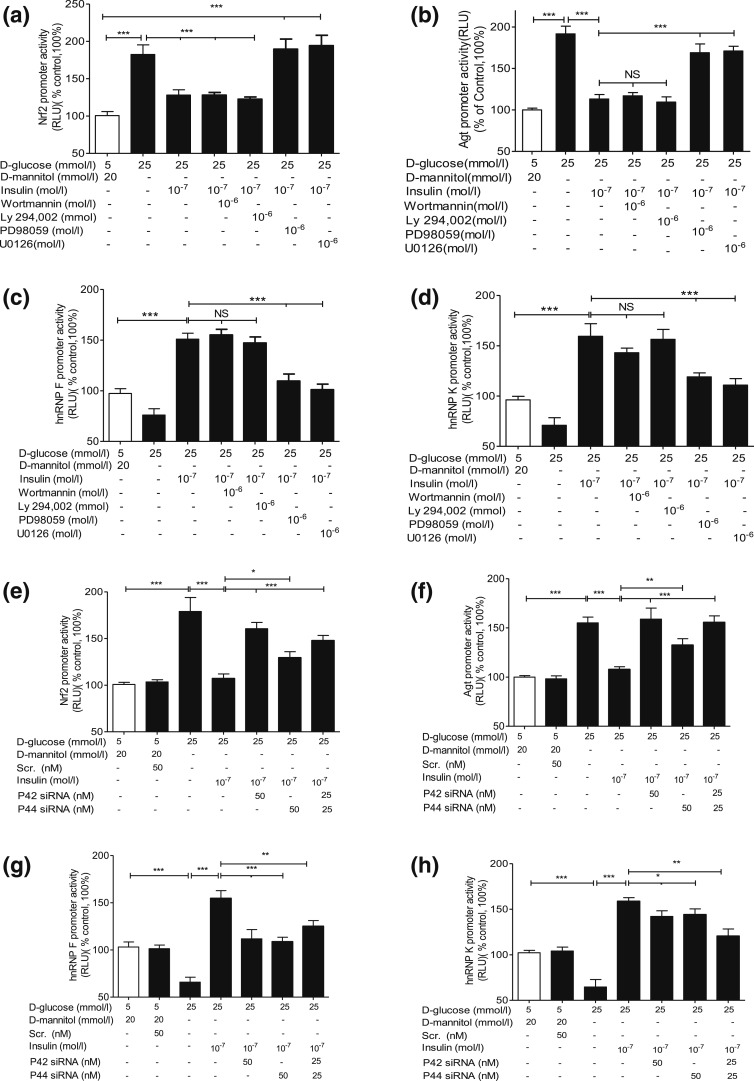
Insulin effect on *Nrf2, Agt, hnRNP F*, and *hnRNP K* gene promoter activity in IRPTCs. Cells stably transfected with (a) pGL4.20-*Nrf2*, (b) pGL4.20-*Agt,* (c) pGL4.20-*hnRNP F,* or (d) pGL4.20-*hnRNP K* gene promoter were incubated in NG or HG DMEM ± insulin for 24 hours with or without wortmannin, Ly-294, 002, PD98059, or U0126 or transiently transfected with p42 MAPK or p44 MAPK siRNA (e–h). Luciferase activity in cells cultured in NG medium was considered as 100%. The results are expressed as percentage of control (mean ± SEM, n = 3). *P < 0.05; **P < 0.01; ***P < 0.005. Similar results were obtained in two separate experiments. NS, not significant; RLU, relative luciferase unit.

Insulin attenuated *Nrf2* and *Agt* gene promoter activity in NG and prevented HG stimulation of *Nrf2* and *Agt* gene promoter activity in IRPTCs in a time-dependent manner [Supplemental Fig. 1(d) and 1(e), respectively]. In contrast, insulin stimulated *hnRNP F* and *hnRNP K* gene promoter activity in NG and HG in IRPTCs in a time-dependent manner [Supplemental Fig. 1(f) and 1(g), respectively]. PD98059 and U0126, but not wortmannin or Ly-294, 002, prevented insulin inhibition of *Nrf2* gene promoter activity [Fig. 3(a)], *Agt* gene promoter activity [Fig. 3(b)], and insulin stimulation of *hnRNP F* [Fig. 3(c)] as well as *hnRNP K* promoter activity [Fig. 3(d)] in IRPTCs.

Insulin stimulated p44/p42 MAPK phosphorylation in a time-dependent manner in NG and HG in IRPTCs [Supplemental Fig. 2(a), i and iii]. Transient transfection of *p44 MAPK* and *p42 MAPK* siRNAs attenuated the expression of respective p44 MAPK and p42 MAPK in IRPTCs, whereas scrambled siRNA had no effect [Supplemental Fig. 3(b)]. Transfection with *p44 MAPK* or *p42 MAPK* siRNAs or both reversed insulin inhibition of *Nrf2* and *Agt* gene promoter activity [Fig. 3(e) and 3(f), respectively] and insulin stimulation of *hnRNP F* and *hnRNP K* gene promoter activity [Fig. 3(g) and 3(h), respectively]. Quantitation of *Nrf2*, *Agt*, *hnRNP F*, and *hnRNP K* mRNA expression [Supplemental Fig. 2(c–f)) confirmed these observations. Our findings lend additional support to the concept that insulin inhibition of *Agt* and *Nrf2* and stimulation of *hnRNP F* and *hnRNP K* transcription require either p44 MAPK or p42 MAPK—or perhaps both—for optimal signaling in RPTCs *in vivo*.

### Insulin prevents Nrf2 stimulation of *Nrf2* and *Agt* gene expression via hnRNP F/K expression in IRPTCs

**Figure 4.**
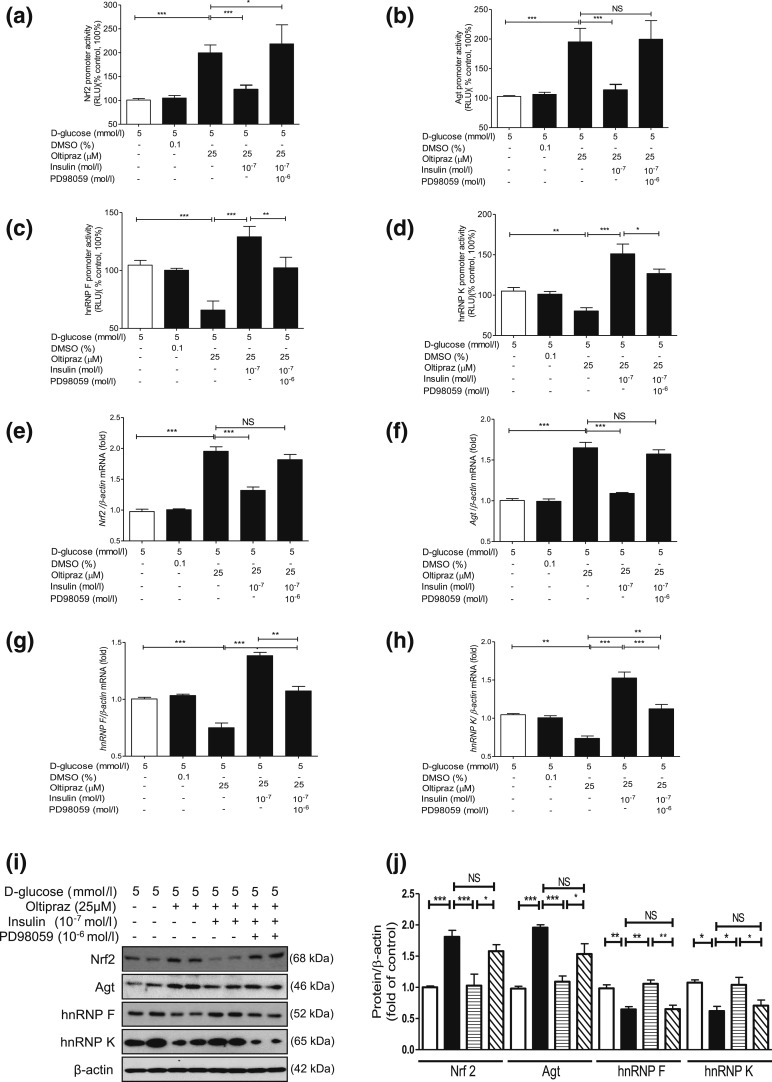
Oltipraz effect on *Agt, Nrf2, hnRNP F*, and *hnRNP K* gene expression in IRPTCs. Effect of oltipraz on promoter activity of (a) *Nrf2,* (b) *Agt,* (c) *hnRNP F*, and (d) *hnRNP K* genes and their respective mRNA (e–h) and protein (i and j) levels in IRPTCs incubated in NG or HG medium ± insulin with or without PD98059. Promoter activity, mRNA, and protein levels in cells in NG medium are considered as 100% or arbitrary unit 1, respectively. The results are reported as percentages of control values (mean ± SEM, n = 3). *P < 0.05; **P < 0.01; ***P < 0.005. Similar results were obtained in three separate experiments. Control IRPTCs in NG (open bars), IRPTCs treated with oltipraz (solid black bars), IRPTCs treated with oltipraz + Ins (horizontal striped bars), and IRPTCs treated with oltipraz + insulin + PD98059 (diagonal striped bars). DMSO, dimethyl sulfoxide; NS, not significant; RLU, relative luciferase unit.

We next explored whether insulin inhibits *Nrf2* gene expression via hnRNP F/K and whether hnRNP F/K could prevent Nrf2 stimulation of *Agt* and *Nrf2* gene transcription in IRPTCs. As anticipated, oltipraz (an Nrf2 activator) stimulated both *Nrf2* and *Agt* gene promoter activity in IRPTCs (11), which was tempered by insulin [Fig. 4(a) and 4(b), respectively]. In contrast, oltipraz diminished *hnRNP F* and *hnRNP K* gene promoter activity that was reversed by insulin [Fig. 4(c) and 4(d), respectively]. Once again, PD98059 reversed these actions of insulin [Fig. 4(a–d)]. Our observations were confirmed by RT-qPCR and WB of their respective mRNA [Fig. 4(e–h)] and protein [Fig. 4(i) and 4(j)] expression.

**Figure 5.**
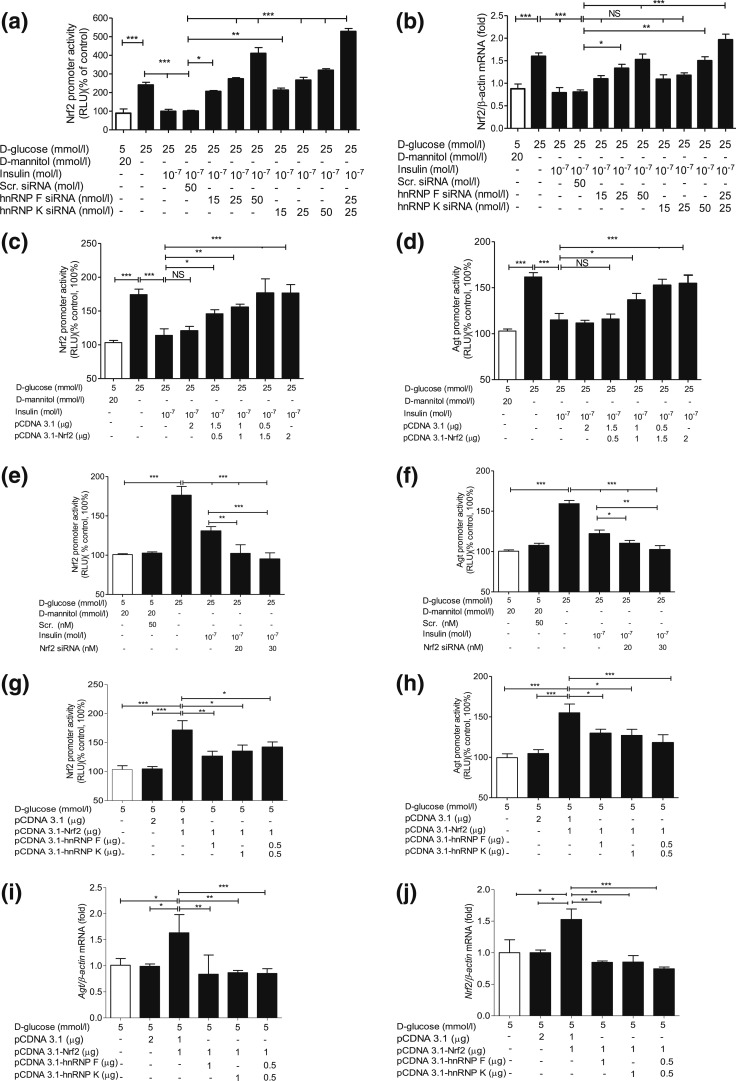
SiRNA of *hnRNP F/K* or *Nrf2* and *hnRNP F/K* cDNA affect *Nrf2* and *Agt* gene promoter activity in IRPTCs. Effect of *hnRNP F* siRNA or *hnRNP K* siRNA or a combination of both on (a) promoter activity and (b) mRNA expression of *Nrf2* in IRPTCs incubated in NG or HG medium ± insulin. Effect of transfection of *Nrf2* cDNA (c and d) and *Nrf2* siRNA (e and f) on *Nrf2* and *Agt* promoter activity and their respective *Nrf2* and *Agt* mRNA levels (g–j) in IRPTCs. Promoter activity and mRNA levels in cells incubated in NG medium are expressed as 100% or arbitrary unit 1, respectively. Each point represents the mean ± SEM (n = 3) assayed in duplicate. *P < 0.05; **P < 0.01; ***P < 0.005. Similar results were obtained in two to three separate experiments. NS, not significant; RLU, relative luciferase unit.

Transfection of *hnRNP F* or *hnRNP K* siRNA or both reversed the inhibitory effect of insulin on *Nrf2* promoter activity and Nrf2 mRNA expression in IRPTCs in HG [Fig. 5(a) and 5(b), respectively], whereas transfection of *Nrf2* complementary DNA (cDNA) attenuated insulin’s inhibitory impact on both *Nrf2* and *Agt* promoter activity in a concentration-dependent manner [Fig. 5(c) and 5(d), respectively]. In contrast, *Nrf2* siRNA transfection further enhanced the suppressive action of insulin on both *Nrf2* and *Agt* gene promoter activity [Fig. 5(e) and 5(f), respectively]. Interestingly, cotransfection with *hnRNP F* and/or *hnRNPK* cDNA tempered the stimulatory effect of *Nrf2* cDNA on *Nrf2* and *Agt* gene promoter activity [Fig. 5(g) and 5(h), respectively] and their mRNA levels [Fig. 5(i) and 5(j), respectively], indicating that hnRNP F and hnRNP K compete with Nrf2 on *Nrf2* and *Agt* gene transcription in RPTCs *in vivo*.

### Localization of hRNP F–responsive elements in rat *Nrf2* gene promoter

**Figure 6.**
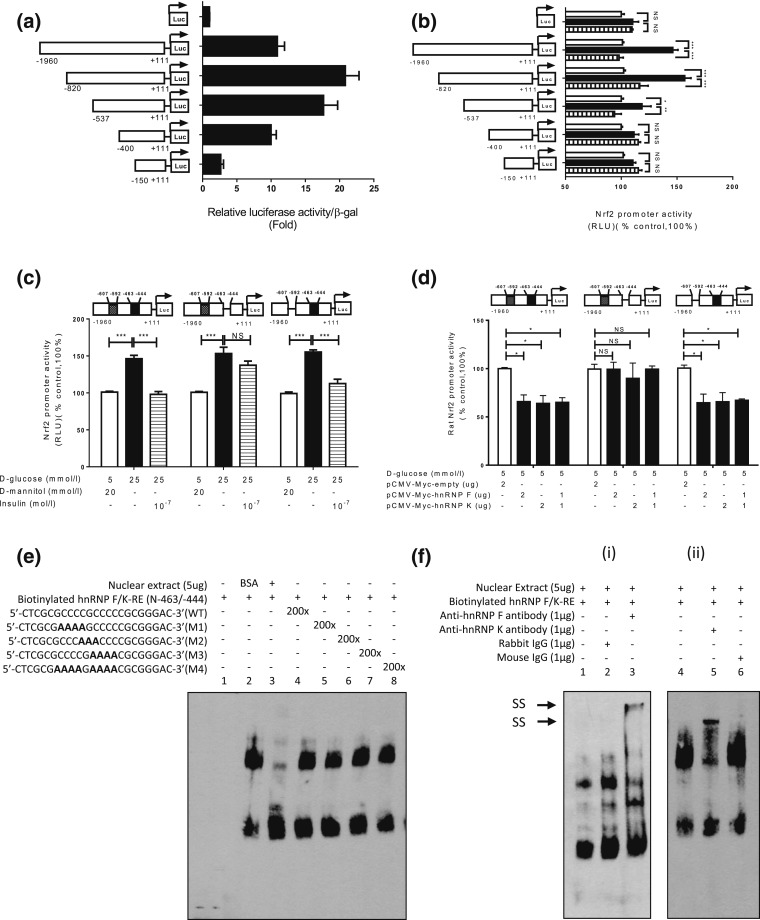
Identification of *hnRNP F/K-RE* or putative *IRE* in the *Nrf2* gene promoter. Luciferase (Luc) activity of plasmids containing various lengths of *Nrf2* gene promoter in (a) NG medium or (b) HG medium ± insulin in IRPTCs. Luciferase activities were normalized by cotransfecting the vector, pRc/RSV plasmid (Invitrogen, Inc.) containing beta-galactosidase cDNA. Control IRPTCs in NG (open bars), IRPTCs in HG (solid black bars), and IRPTCs treated with Ins in HG (horizontal striped bars). (c) Activity of 1 μg of the full-length *Nrf2* gene promoter ± deletion of distal putative *IRE* (*N*-607 to *N*-592; 5′-ggggcccgggctccc-3′) or proximal putative *IRE* (*N*-463 to *N*-444 (5′-cgcgccccgcccccgcggga-3″) in IRPTCs in NG medium. (d) Activity of 1 μg of the full-length *Nrf2* gene promoter with or without deletion of distal putative *IRE* or proximal putative *IRE* transfected with *hnRNP F* or *hnRNP K* cDNA or a combination of both in IRPTCs in NG medium. Values are mean ± SEM, n = 3. All experiments were repeated twice. (*P < 0.05; **P < 0.01; ***P < 0.005). (e) EMSA of putative biotinylated proximal *IRE* with RPTC nuclear proteins with or without excess unlabeled proximal WT *IRE* or mutated *IRE*. (f) Supershift EMSA. (i) Rabbit anti–hnRNP F or rabbit IgG and (ii) mouse anti–hnRNP K or mouse IgG was added to the reaction mixture and incubated for 30 minutes on ice before incubation with biotinylated probe. The results are representative of three independent experiments. NS, not significant; RLU, relative luciferase unit; SS, supershift band.

To localize putative DNA-responsive elements (REs) that mediate insulin’s inhibitory action, plasmids containing various lengths of the rat *Nrf2* gene promoter were transiently transfected into IRPTCs. pGL4.20-*Nrf2* promoter *N*-1960/N+111 exhibited 10-fold increases compared with control plasmid promoterless pGL4.20 in IRPTCs [Fig. 6(a)]. Deletion of nucleotides to *N*-820, *N*-537, and *N*-400 augmented the activity of pGL4.20-*Nrf2* promoter *N*-820/N+111, pGL4.20-*Nrf2* promoter *N*-537/N+111, and pGL4.20-*Nrf2* promoter *N*-400/N+111 to 22-, 18-, and 10-fold compared with control plasmid pGL4.20, respectively. Furthermore, deletion of nucleotides to *N*-150 lowered the promoter activity of pGL4.20-*Nrf2* promoter *N*-150/+111 to 2.5-fold higher than the control [Fig. 6(a)]. Insulin averted the stimulatory effect of HG on pGL4.20-*Nrf2* promoters *N*-1960/N+111, *N*-820/N+111 and *N*-537/N+111, whereas HG and insulin had no impact on the activity of the pGL4.20-*Nrf2* promoters *N*-400/N+111 and *N*-150/N+111 [Fig. 6(b)]. Interestingly, deletion of nucleotides *N*-463 to *N*-444 (5′-cgcgccccgcccccgcggga-3′) in the *Nrf2* gene promoter completely abolished the inhibitory action of insulin on pGL4.20-*Nrf2* promoter *N*-1960/N+111 activity in HG, whereas deletion of *N*-607 to *N*-592 (5′-ggggcccgggctccc-3′) in the *Nrf2* gene promoter had no effect [Fig. 6(c)]. Furthermore, transfection of the plasmid pCMV-Myc containing *hnRNP F* or *hnRNP K* cDNA or both plasmids inhibited pGL4.20 *Nrf2* promoter *N*-1960/N+111 activity with or without *N*-607 to *N*-592 deletion, but had no impact on *Nrf2* gene promoter activity with *N*-463 to *N*-444 deletion [Fig. 6(d)]. These data would point toward nucleotides *N*-463 to *N*-444 as a putative *IRE* that binds hnRNP F/K.

The EMSA showed that the double-strand DNA fragment, *N*-465 to *N*-443 (WT), binds to nuclear proteins from IRPTCs and could be displaced by the respective WT DNA fragment, but not by mutated DNA fragments [Fig. 6(e)]. Furthermore, addition of anti-hnRNP F or anti-hnRNP K antibody induced a supershift of the *hnRNP F-*responsive element (*RE*) with nuclear proteins [Fig. 6(f), i and ii, respectively].

### Oxidative stress and gene expression in hyperinsulinemic-euglycemic mouse kidneys

**Figure 7.**
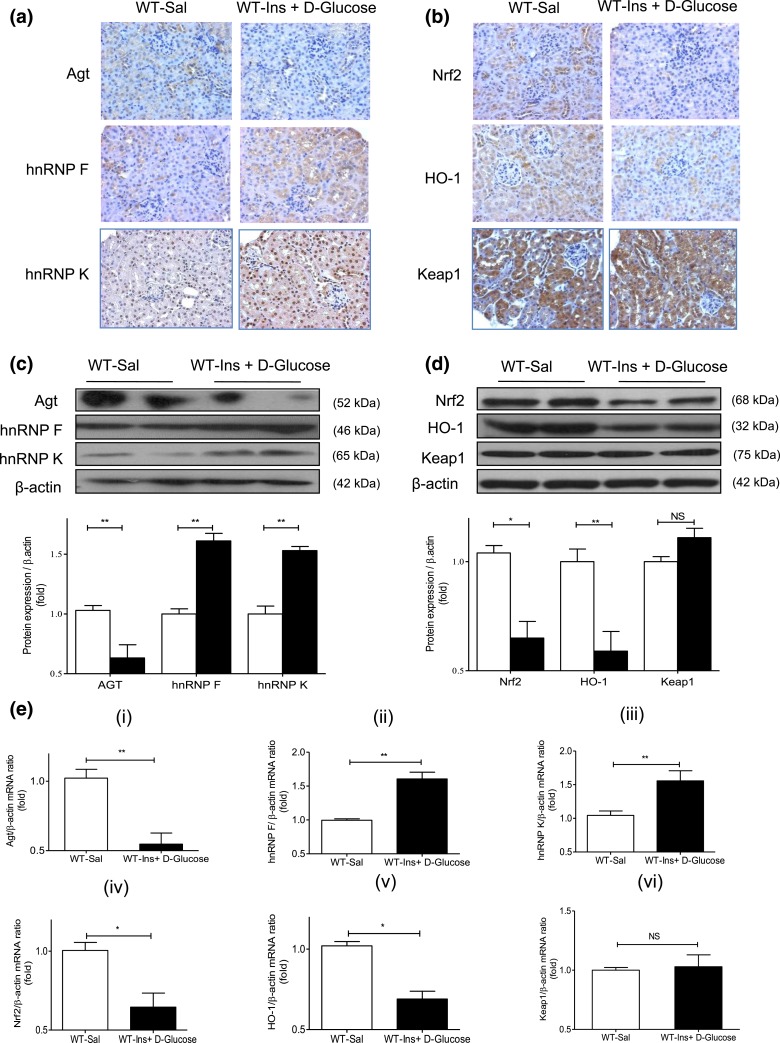
Renal Agt, hnRNP F/K, Nrf2, HO-1, and Keap1 expression in hyperinsulinemic-euglycemic mice. (a) Immunostaining of Agt, hnRNP F, and hnRNP K and (b) Nrf2, HO-1, and Keap1 (magnification ×200). (c) WB of Agt, hnRNP F, and hnRNP K and (d) Nrf2, HO-1, and Keap1 expression in isolated RPTs from WT mice after a 3-hour infusion with saline (Sal) or insulin (Ins) + d-glucose. (e) RT-qPCR of (i) *Agt*, (ii) *hnRNP F*, (iii) *hnRNP K,* (iv) *Nrf2,* (v) *HO-1*, and (vi) *Keap1* mRNA expression in isolated RPTs from WT mice after a 3-hour infusion with saline (Sal; open bars) or Ins + d-glucose (solid black bars). Values are mean ± SEM, n = 8 per group. *P < 0.05; **P < 0.01. NS, not significant.

To investigate whether insulin could influence renal *Agt*, *Nrf2*, *hnRNP F*, and *hnRNP K* expression independently of its glucose-lowering effect *in vivo*, we performed hyperinsulinemic-euglycemic clamp experiments on WT mice [Supplemental Fig. 3(a–c)]. DHE staining, ROS generation, *Cat, Nox1, Nox2*, and *Nox4* mRNA expression [Supplemental Fig. 3(d–i)] did not differ from RPTs of saline-infused and hyperinsulinemic mice. In contrast, hyperinsulinemia decreased Agt and increased hnRNP F and hnRNP K immunostaining [Fig. 7(a)]. It also reduced Nrf2 and HO-1 immunostaining without affecting Keap1 compared with saline infusion [Fig. 7(b)]. WB [Fig. 7(c) and 7(d)] and RT-qPCR [Fig. 7(e), i–iv] of their respective protein and mRNA expressions confirmed these findings.

## Discussion

Our present study identifies an inhibitory action of insulin on renal *Nrf2* gene transcription via a putative *IRE* in the *Nrf2* gene promoter that binds hnRNP F/K. Insulin also prevents *Nrf2* stimulation of *Agt* expression via hnRNP F/K expression in diabetes. These insulin-mediated effects largely occur independently of its glucose-lowering effect.

Intensive insulin therapy is critical for preventing the progression of nephropathy in T1D, although the underlying mechanisms remain incompletely understood (31–33). The existence of a local RAS in the kidney is well-established (34, 35). RPTCs express all components of the RAS (29, 36, 37). We demonstrated previously that insulin prevents hypertension and attenuates kidney injury by suppressing renal *Agt* gene transcription via hnRNP F/K upregulation in Akita mice (23). The current study provides *in vivo* and *in vitro* evidence that insulin modulates *Agt* expression more proximally; it curtails renal *Nrf2* gene transcription and prevents Nrf2 stimulation of *Agt* expression by increasing hnRNP F/K expression, which may be critical for its antihypertensive and renoprotective actions in diabetes.

The Akita mouse, an autosomal-dominant model of spontaneous T1D (*insulin2* mutation), develops hypoinsulinemia (60% to 70% lower circulating immunoreactive insulin levels), hyperglycemia, hypertension, cardiac, and renal dysfunction (38, 39) closely resembling changes in T1D patients. We detected markedly increased oxidative stress in RPTCs from Akita compared with non-Akita mice; insulin normalized these changes. Consistently, insulin treatment lowered RPT *Nrf2* and *Agt* expression as well as urinary Agt and Ang II levels in Akita mice vs WT controls. Thus, the Akita mouse is an excellent model of T1D with insulin repletion.

Cat expression and activity, but not *Cat* mRNA expression, were significantly lower in RPTs from Akita vs WT mice at 16 weeks of age. In contrast, no substantial changes in Cat expression and activity were detected in RPTs of younger Akita mice (4 weeks of age) when compared with WT mice [Supplemental Fig. 4(c–f)], leading us to speculate that the lower Cat expression and activity observed in Akita mice at 16 weeks of age might be due to exhaustion of the scavenging system.

Interestingly, treatment of Akita mice with insulin implants at 20 weeks of age markedly attenuated SBP, fasting blood glucose, KW–body weight ratio and KW/TL, (with the exception of urinary ACR), normalized *Nrf2*, and *Agt* mRNA expression and stimulated p44/42 MAPK phosphorylation in RPTs of Akita mice at 24 weeks [Supplemental Table 1 and Supplemental Fig. 5(a–c)]. These findings are consistent with those of Lizotte *et al.* (40), who reported that insulin treatment was effective in lowering fasting blood glucose, but not urinary ACR in Akita mice when begun at the age of 20 weeks. However, whether insulin is effective in even older Akita mice remains to be investigated.

The insulin level used *in vitro* (10^−7^ M or 573 ng/mL) was at least 200-fold higher than the mean circulating insulin level in Akita mice bearing insulin implants (2.3 ± 1.1 ng/mL), similar to those reported (3.4 ± 0.4 ng/mL) by others (41). However, we routinely used insulin at 10^−7^ M for our *in vitro* studies because we found that insulin at 10^−7^ M completely normalized *Nrf2* and *Agt* promoter activity and enhanced *hnRNP F/K* promoter activity 1.5-fold compared with insulin at 10^−9^ M in HG [Supplemental Fig. 6(a–d)].

Combining pharmacological inhibitors and gene knockdown with siRNAs, we identified a key role of the p44/42 MAPK pathway mediating insulin suppression of renal *Nrf2* and *Agt* as well as stimulation of *hnRNP F/K* gene transcription. At present, we do not understand the exact mechanism by which insulin decreases nuclear Nrf2 accumulation in Akita mice. Studies of Zheng *et al.* (42), which reported that mutation of consensus sites (s215, s408, and s577) for MAPK phosphorylation in Nrf2 by MAPKs had a limited impact in mediating Nrf2 nuclear translocation and activity in HEK293T cells. One possibility is that insulin activates p44/42 MAPK following binding to insulin receptors (23, 43, 44), then phosphorylates Nrf2, thereby modulating or hindering their nuclear translocation and activity. This possibility is supported by our data, which show that insulin treatment attenuates nuclear accumulation of Nrf2 and *p*-Nrf2 (s-40) without apparent effect on cytoplasmic Nrf2 and *p*-Nrf2 (s-40) in Akita mice and increases p44/42 MAPK phosphorylation [Supplemental Fig. 4(a) and 4(b), respectively]. During oxidative stress, PKC-*δ* phosphorylates Nrf2 at serine 40 to enhance its nuclear translocation (45, 46). Another possibility is that p44/42 could directly affect *Agt, Nrf2*, and *hnRNP F/K* transcription via binding to the putative MAPK-responsive element(s) in the respective promoters. Hu *et al.* (47) reported that MAPK1 could act as a transcriptional repressor for interferon gamma-induced genes via binding to a G/C AAA G/C consensus sequence. Clearly, additional studies along these lines are required to elucidate the mechanisms underlying the effects of p44/42 MAPK on *Agt, Nrf2*, and *hnRNP F/K* transcription.

Interestingly, Nrf2 overexpression prevented—whereas Nrf2 siRNA enhanced—insulin inhibition of *Nrf2* and *Agt* gene transcription in IRPTCs. These effects could be explained by the presence of *Nrf2-RE* in both *Nrf2* (48) and *Agt* (11) promoters. Nrf2 may exert a positive auto-feedback on *Nrf2* transcription (48).

The precise mechanism by which hnRNP F/K mediate insulin downregulation of renal *Nrf2* gene expression in diabetes remains unclear. One possibility is that hnRNP F/K bind to putative *DNA-RE* (tentatively designated as “*IRE*”) in *Nrf2* gene promoter, subsequently suppressing *Nrf2* gene transcription. This possibility is supported by our finding that hnRNP F/K overexpression considerably decreases *Nrf2* gene promoter activity, and *hnRNP F/K* siRNA reverse insulin downregulation of *Nrf2* gene transcription. DNA sequence analysis discerned 2 GC-rich regions, nucleotides *N*-463 to *N*-444 (5′-cgcgccccgcccccgcggga-3′) and *N*-607 to *N*-592 (5′-ggggcccgggctccc-3′), in the *Nrf2* gene promoter. Nucleotides *N*-463 to *N*-444 contain the core sequence 5′-**ccccgcccc**-3′, which is homologous to the core sequence of *IRE* (*N*-882 to *N*-855; 5′-cctccct**tcccgccct**tcactttctagt-3′) of the rat *Agt* gene promoter (20, 21). Deletion of *N*-463 to *N*-444, but not *N*-607 to *N*-592, in the *Nrf2* gene promoter markedly reduces insulin- and hnRNP F/K-downregulation of *Nrf2* gene promoter activity in IRPTCs. Moreover, biotinylated-labeled *IRE* (*N*-463 to *N*-444) specifically binds to RPTC nuclear proteins, and the addition of antihnRNP F or antihnRNP K antibody yields a supershift of biotinylated-labeled *IRE* binding with nuclear proteins on EMSA. These data demonstrate that hnRNP F/K bind to a putative *IRE* (*N*-463 to *N*-444) and inhibit *Nrf2* gene transcription. It is noteworthy that hnRNP F/K are not restricted to *Nrf2* gene expression but also affect the expression of *Agt* (20, 21), *Ace2* (30), and other genes (49, 50).

In RPTCs of hyperinsulinemic-euglycemic mice, insulin suppressed *Agt*, *Nrf2*, and *HO-1* expression and stimulated *hnRNP F/K* expression. Its effect was rapid (3 hours after hyperinsulinemia) compared with insulin implants in Akita mice (after 4 weeks of insulin implantation). Such rapid transcription is consistent with other studies of upregulated and downregulated genes in muscles and liver within 2 to 4 hours under euglycemic-hyperinsulinemic conditions (14, 15). This would indicate that insulin could directly impact renal *Nrf2* and *Agt* gene expression, in addition to its glucose-lowering action.

Finally, *post hoc* analysis of bardoxolone methyl failure in the BEACON trial suggests that the adverse effects in treated patients might be mediated through the endothelin 1 pathway (51, 52). It has been noted (53), however, that bardoxolone methyl heightened SBP and worsened albuminuria, whereas selective ET-A antagonists lessened them in the Efficacy and Safety of Pirfenidone in Patients With Idiopathic Pulmonary Fibrosis trial (54). Our study demonstrates that insulin treatment prevents oltipraz and Nrf2 stimulation of *Agt* gene expression, suggesting that chronic Nrf2 activation by hyperglycemia and/or Nrf2 activator(s) may exaggerate renal dysfunction via activation of the intrarenal RAS, thereby enhancing renal fluid and salt reabsorption.

In summary, our data demonstrate that insulin inhibits *Nrf2* gene transcription and prevents Nrf2 stimulation of intrarenal *Agt* gene expression via hnRNP F/K, indicating that Nrf2 activation may amplify renal dysfunction via intrarenal RAS activation in diabetes. Our study identifies renal hnRNP F/K and Nrf2 as potential targets for the treatment of hypertension and kidney injury in diabetes.

## References

[1] Venugopal R, Jaiswal AK Nrf1 and Nrf2 positively and c-Fos and Fra1 negatively regulate the human antioxidant response element-mediated expression of NAD(P)H:quinone oxidoreductase1 gene. Proc Natl Acad Sci USA. 1996;93(25):14960–14965. 8962164 10.1073/pnas.93.25.14960 PMC26245

[2] Motohashi H, Yamamoto M Nrf2-Keap1 defines a physiologically important stress response mechanism. Trends Mol Med. 2004;10(11):549–557. 15519281 10.1016/j.molmed.2004.09.003

[3] Surh YJ, Kundu JK, Na HK Nrf2 as a master redox switch in turning on the cellular signaling involved in the induction of cytoprotective genes by some chemopreventive phytochemicals. Planta Med. 2008;74(13):1526–1539. 18937164 10.1055/s-0028-1088302

[4] Jiang T, Huang Z, Lin Y, Zhang Z, Fang D, Zhang DD The protective role of Nrf2 in streptozotocin-induced diabetic nephropathy. Diabetes. 2010;59(4):850–860. 20103708 10.2337/db09-1342 PMC2844833

[5] Zheng H, Whitman SA, Wu W, Wondrak GT, Wong PK, Fang D, Zhang DD Therapeutic potential of Nrf2 activators in streptozotocin-induced diabetic nephropathy. Diabetes. 2011;60(11):3055–3066. 22025779 10.2337/db11-0807 PMC3198067

[6] Tan SM, Sharma A, Stefanovic N, Yuen DY, Karagiannis TC, Meyer C, Ward KW, Cooper ME, de Haan JB Derivative of bardoxolone methyl, dh404, in an inverse dose-dependent manner lessens diabetes-associated atherosclerosis and improves diabetic kidney disease. Diabetes. 2014;63(9):3091–3103. 24740568 10.2337/db13-1743

[7] Yore MM, Liby KT, Honda T, Gribble GW, Sporn MB The synthetic triterpenoid 1-[2-cyano-3, 12-dioxooleana-1, 9(11)-dien-28-oyl]imidazole blocks nuclear factor-kappaB activation through direct inhibition of IkappaB kinase beta. Mol Cancer Ther. 2006;5(12):3232–3239. 17148759 10.1158/1535-7163.MCT-06-0444

[8] Liby K, Voong N, Williams CR, Risingsong R, Royce DB, Honda T, Gribble GW, Sporn MB, Letterio JJ. The synthetic triterpenoid CDDO-Imidazolide suppresses STAT phosphorylation and induces apoptosis in myeloma and lung cancer cells. *Clin Cancer Res*. 2006;**12**:4288–4293. 10.1158/1078-0432.CCR-06-0215 16857804

[9] Pergola PE, Raskin P, Toto RD, Meyer CJ, Huff JW, Grossman EB, Krauth M, Ruiz S, Audhya P, Christ-Schmidt H, Wittes J, Warnock DG; BEAM Study Investigators Bardoxolone methyl and kidney function in CKD with type 2 diabetes. N Engl J Med. 2011;365(4):327–336. 21699484 10.1056/NEJMoa1105351

[10] de Zeeuw D, Akizawa T, Audhya P, Bakris GL, Chin M, Christ-Schmidt H, Goldsberry A, Houser M, Krauth M, Lambers Heerspink HJ, McMurray JJ, Meyer CJ, Parving HH, Remuzzi G, Toto RD, Vaziri ND, Wanner C, Wittes J, Wrolstad D, Chertow GM; BEACON Trial Investigators Bardoxolone methyl in type 2 diabetes and stage 4 chronic kidney disease. N Engl J Med. 2013;369(26):2492–2503. 24206459 10.1056/NEJMoa1306033 PMC4496027

[11] Abdo S, Shi Y, Otoukesh A, Ghosh A, Lo CS, Chenier I, Filep JG, Ingelfinger JR, Zhang SL, Chan JS Catalase overexpression prevents nuclear factor erythroid 2-related factor 2 stimulation of renal angiotensinogen gene expression, hypertension, and kidney injury in diabetic mice. Diabetes. 2014;63(10):3483–3496. 24812425 10.2337/db13-1830 PMC4171660

[12] Brezniceanu ML, Liu F, Wei CC, Chénier I, Godin N, Zhang SL, Filep JG, Ingelfinger JR, Chan JS Attenuation of interstitial fibrosis and tubular apoptosis in db/db transgenic mice overexpressing catalase in renal proximal tubular cells. Diabetes. 2008;57(2):451–459. 17977949 10.2337/db07-0013

[13] Shi Y, Lo CS, Chenier I, Maachi H, Filep JG, Ingelfinger JR, Zhang SL, Chan JS Overexpression of catalase prevents hypertension and tubulointerstitial fibrosis and normalization of renal angiotensin-converting enzyme-2 expression in Akita mice. Am J Physiol Renal Physiol. 2013;304(11):F1335–F1346. 23552863 10.1152/ajprenal.00405.2012 PMC3680689

[14] Coletta DK, Balas B, Chavez AO, Baig M, Abdul-Ghani M, Kashyap SR, Folli F, Tripathy D, Mandarino LJ, Cornell JE, Defronzo RA, Jenkinson CP Effect of acute physiological hyperinsulinemia on gene expression in human skeletal muscle in vivo. Am J Physiol Endocrinol Metab. 2008;294(5):E910–E917. 18334611 10.1152/ajpendo.00607.2007 PMC3581328

[15] Korsheninnikova E, Voshol PJ, Baan B, van der Zon GC, Havekes LM, Romijn JA, Maassen JA, Ouwens DM Dynamics of insulin signalling in liver during hyperinsulinemic euglycaemic clamp conditions in vivo and the effects of high-fat feeding in male mice. Arch Physiol Biochem. 2007;113(4-5):173–185. 18158643 10.1080/13813450701669084

[16] Chen X, Zhang SL, Pang L, Filep JG, Tang SS, Ingelfinger JR, Chan JS Characterization of a putative insulin-responsive element and its binding protein(s) in rat angiotensinogen gene promoter: regulation by glucose and insulin. Endocrinology. 2001;142(6):2577–2585. 11356707 10.1210/endo.142.6.8214

[17] Hsieh TJ, Fustier P, Wei CC, Zhang SL, Filep JG, Tang SS, Ingelfinger JR, Fantus IG, Hamet P, Chan JS Reactive oxygen species blockade and action of insulin on expression of angiotensinogen gene in proximal tubular cells. J Endocrinol. 2004;183(3):535–550. 15590980 10.1677/joe.1.05871

[18] Hsieh TJ, Fustier P, Zhang SL, Filep JG, Tang SS, Ingelfinger JR, Fantus IG, Hamet P, Chan JS High glucose stimulates angiotensinogen gene expression and cell hypertrophy via activation of the hexosamine biosynthesis pathway in rat kidney proximal tubular cells. Endocrinology. 2003;144(10):4338–4349. 12960040 10.1210/en.2003-0220

[19] Hsieh TJ, Zhang SL, Filep JG, Tang SS, Ingelfinger JR, Chan JS High glucose stimulates angiotensinogen gene expression via reactive oxygen species generation in rat kidney proximal tubular cells. Endocrinology. 2002;143(8):2975–2985. 12130563 10.1210/endo.143.8.8931

[20] Wei CC, Guo DF, Zhang SL, Ingelfinger JR, Chan JS Heterogenous nuclear ribonucleoprotein F modulates angiotensinogen gene expression in rat kidney proximal tubular cells. J Am Soc Nephrol. 2005;16(3):616–628. 15659559 10.1681/ASN.2004080715

[21] Wei CC, Zhang SL, Chen YW, Guo DF, Ingelfinger JR, Bomsztyk K, Chan JS Heterogeneous nuclear ribonucleoprotein K modulates angiotensinogen gene expression in kidney cells. J Biol Chem. 2006;281(35):25344–25355. 16837467 10.1074/jbc.M601945200

[22] Lo CS, Chang SY, Chenier I, Filep JG, Ingelfinger JR, Zhang SL, Chan JS Heterogeneous nuclear ribonucleoprotein F suppresses angiotensinogen gene expression and attenuates hypertension and kidney injury in diabetic mice. Diabetes. 2012;61(10):2597–2608. 22664958 10.2337/db11-1349 PMC3447919

[23] Abdo S, Lo CS, Chenier I, Shamsuyarova A, Filep JG, Ingelfinger JR, Zhang SL, Chan JS Heterogeneous nuclear ribonucleoproteins F and K mediate insulin inhibition of renal angiotensinogen gene expression and prevention of hypertension and kidney injury in diabetic mice. Diabetologia. 2013;56(7):1649–1660. 23609310 10.1007/s00125-013-2910-4

[24] Chan JS, Chan AH, Jiang Q, Nie ZR, LaChance S, Carrière S Molecular cloning and expression of the rat angiotensinogen gene. Pediatr Nephrol. 1990;4(4):429–435. 2206913 10.1007/BF00862531

[25] Wang L, Lei C, Zhang SL, Roberts KD, Tang SS, Ingelfinger JR, Chan JS Synergistic effect of dexamethasone and isoproterenol on the expression of angiotensinogen in immortalized rat proximal tubular cells. Kidney Int. 1998;53(2):287–295. 9461088 10.1046/j.1523-1755.1998.00759.x

[26] Lo CS, Liu F, Shi Y, Maachi H, Chenier I, Godin N, Filep JG, Ingelfinger JR, Zhang SL, Chan JS Dual RAS blockade normalizes angiotensin-converting enzyme-2 expression and prevents hypertension and tubular apoptosis in Akita angiotensinogen-transgenic mice. Am J Physiol Renal Physiol. 2012;302(7):F840–F852. 22205225 10.1152/ajprenal.00340.2011 PMC3340935

[27] Alquier T, Peyot ML, Latour MG, Kebede M, Sorensen CM, Gesta S, Ronald Kahn C, Smith RD, Jetton TL, Metz TO, Prentki M, Poitout V Deletion of GPR40 impairs glucose-induced insulin secretion in vivo in mice without affecting intracellular fuel metabolism in islets. Diabetes. 2009;58(11):2607–2615. 19720802 10.2337/db09-0362 PMC2768167

[28] Godin N, Liu F, Lau GJ, Brezniceanu ML, Chénier I, Filep JG, Ingelfinger JR, Zhang SL, Chan JS Catalase overexpression prevents hypertension and tubular apoptosis in angiotensinogen transgenic mice. Kidney Int. 2010;77(12):1086–1097. 20237455 10.1038/ki.2010.63

[29] Tang SS, Jung F, Diamant D, Brown D, Bachinsky D, Hellman P, Ingelfinger JR Temperature-sensitive SV40 immortalized rat proximal tubule cell line has functional renin-angiotensin system. Am J Physiol. 1995;268(3 Pt 2):F435–F446. 7900843 10.1152/ajprenal.1995.268.3.F435

[30] Lo CS, Shi Y, Chang SY, Abdo S, Chenier I, Filep JG, Ingelfinger JR, Zhang SL, Chan JS Overexpression of heterogeneous nuclear ribonucleoprotein F stimulates renal Ace-2 gene expression and prevents TGF-β1-induced kidney injury in a mouse model of diabetes. Diabetologia. 2015;58(10):2443–2454. 26232095 10.1007/s00125-015-3700-y PMC4572079

[31] The Diabetes Control and Complications Trial Research Group The effect of intensive treatment of diabetes on the development and progression of long-term complications in insulin-dependent diabetes mellitus. N Engl J Med. 1993;329(14):977–986. 8366922 10.1056/NEJM199309303291401

[32] The Diabetes Control and Complications Trial/Epidemiology of Diabetes Interventions and Complications Research Group Retinopathy and nephropathy in patients with type 1 diabetes four years after a trial of intensive therapy. N Engl J Med. 2000;342(6):381–389. 10666428 10.1056/NEJM200002103420603 PMC2630213

[33] de Boer IH, Rue TC, Cleary PA, Lachin JM, Molitch ME, Steffes MW, Sun W, Zinman B, Brunzell JD, White NH, Danis RP, Davis MD, Hainsworth D, Hubbard LD, Nathan DM; Diabetes Control and Complications Trial/Epidemiology of Diabetes Interventions and Complications Study Research Group Long-term renal outcomes of patients with type 1 diabetes mellitus and microalbuminuria: an analysis of the Diabetes Control and Complications Trial/Epidemiology of Diabetes Interventions and Complications cohort. Arch Intern Med. 2011;171(5):412–420. 21403038 10.1001/archinternmed.2011.16 PMC3085024

[34] Dzau VJ, Ingelfinger JR. Molecular biology and pathophysiology of the intrarenal renin-angiotensin system. *J Hypertens Suppl*. 1989;**7**:S3–S8. 10.1097/00004872-198909007-00002 2559171

[35] Johnston CI, Fabris B, Jandeleit K Intrarenal renin-angiotensin system in renal physiology and pathophysiology. Kidney Int Suppl. 1993;42:S59–S63. 8361131

[36] Loghman-Adham M, Rohrwasser A, Helin C, Zhang S, Terreros D, Inoue I, Lalouel JM A conditionally immortalized cell line from murine proximal tubule. Kidney Int. 1997;52(1):229–239. 9211368 10.1038/ki.1997.325

[37] Wolf G, Neilson EG Angiotensin II as a hypertrophogenic cytokine for proximal tubular cells. Kidney Int Suppl. 1993;39:S100–S107. 8468911

[38] Wang J, Takeuchi T, Tanaka S, Kubo SK, Kayo T, Lu D, Takata K, Koizumi A, Izumi T A mutation in the insulin 2 gene induces diabetes with severe pancreatic beta-cell dysfunction in the Mody mouse. J Clin Invest. 1999;103(1):27–37. 9884331 10.1172/JCI4431 PMC407861

[39] Yoshioka M, Kayo T, Ikeda T, Koizumi A A novel locus, Mody4, distal to D7Mit189 on chromosome 7 determines early-onset NIDDM in nonobese C57BL/6 (Akita) mutant mice. Diabetes. 1997;46(5):887–894. 9133560 10.2337/diab.46.5.887

[40] Lizotte F, Denhez B, Guay A, Gévry N, Côté AM, Geraldes P Persistent insulin resistance in podocytes caused by epigenetic change of SHP-1 in diabetes. Diabetes. 2016;65(12):3705–3717. 27585521 10.2337/db16-0254

[41] Salem ESB, Grobe N, Elased KM Insulin treatment attenuates renal ADAM17 and ACE2 shedding in diabetic Akita mice. Am J Physiol Renal Physiol. 2014;306(6):F629–F639. 24452639 10.1152/ajprenal.00516.2013 PMC3949038

[42] Sun Z, Huang Z, Zhang DD Phosphorylation of Nrf2 at multiple sites by MAP kinases has a limited contribution in modulating the Nrf2-dependent antioxidant response. PLoS One. 2009;4(8):e6588. 19668370 10.1371/journal.pone.0006588 PMC2719090

[43] Zhang SL, Chen X, Filep JG, Tang SS, Ingelfinger JR, Chan JS Insulin inhibits angiotensinogen gene expression via the mitogen-activated protein kinase pathway in rat kidney proximal tubular cells. Endocrinology. 1999;140(11):5285–5292. 10537159 10.1210/endo.140.11.7125

[44] Zhang SL, Chen X, Wei CC, Filep JG, Tang SS, Ingelfinger JR, Chan JS Insulin inhibits dexamethasone effect on angiotensinogen gene expression and induction of hypertrophy in rat kidney proximal tubular cells in high glucose. Endocrinology. 2002;143(12):4627–4635. 12446590 10.1210/en.2002-220408

[45] Huang HC, Nguyen T, Pickett CB Phosphorylation of Nrf2 at Ser-40 by protein kinase C regulates antioxidant response element-mediated transcription. J Biol Chem. 2002;277(45):42769–42774. 12198130 10.1074/jbc.M206911200

[46] Niture SK, Jain AK, Jaiswal AK Antioxidant-induced modification of INrf2 cysteine 151 and PKC-δ-mediated phosphorylation of Nrf2 serine 40 are both required for stabilization and nuclear translocation of Nrf2 and increased drug resistance. J Cell Sci. 2009;122(Pt 24):4452–4464. 19920073 10.1242/jcs.058537 PMC2787459

[47] Hu S, Xie Z, Onishi A, Yu X, Jiang L, Lin J, Rho HS, Woodard C, Wang H, Jeong J-S, Long S, He X, Wade H, Blackshaw S, Qian J, Zhu H Profiling the human protein-DNA interactome reveals ERK2 as a transcriptional repressor of interferon signaling. Cell. 2009;139(3):610–622. 19879846 10.1016/j.cell.2009.08.037 PMC2774939

[48] Kwak MK, Itoh K, Yamamoto M, Kensler TW Enhanced expression of the transcription factor Nrf2 by cancer chemopreventive agents: role of antioxidant response element-like sequences in the nrf2 promoter. Mol Cell Biol. 2002;22(9):2883–2892. 11940647 10.1128/MCB.22.9.2883-2892.2002 PMC133753

[49] Chen Y, Schnetz MP, Irarrazabal CE, Shen RF, Williams CK, Burg MB, Ferraris JD Proteomic identification of proteins associated with the osmoregulatory transcription factor TonEBP/OREBP: functional effects of Hsp90 and PARP-1. Am J Physiol Renal Physiol. 2007;292(3):F981–F992. 17148781 10.1152/ajprenal.00493.2005

[50] Wang E, Aslanzadeh V, Papa F, Zhu H, de la Grange P, Cambi F Global profiling of alternative splicing events and gene expression regulated by hnRNPH/F. PLoS One. 2012;7(12):e51266. 23284676 10.1371/journal.pone.0051266 PMC3524136

[51] Chin MP, Reisman SA, Bakris GL, O’Grady M, Linde PG, McCullough PA, Packham D, Vaziri ND, Ward KW, Warnock DG, Meyer CJ Mechanisms contributing to adverse cardiovascular events in patients with type 2 diabetes mellitus and stage 4 chronic kidney disease treated with bardoxolone methyl. Am J Nephrol. 2014;39(6):499–508. 24903467 10.1159/000362906

[52] Chin MP, Wrolstad D, Bakris GL, Chertow GM, de Zeeuw D, Goldsberry A, Linde PG, McCullough PA, McMurray JJ, Wittes J, Meyer CJ Risk factors for heart failure in patients with type 2 diabetes mellitus and stage 4 chronic kidney disease treated with bardoxolone methyl. J Card Fail. 2014;20(12):953–958. 25307295 10.1016/j.cardfail.2014.10.001

[53] Van Laecke S, Van Biesen W, Vanholder R The paradox of bardoxolone methyl: a call for every witness on the stand? Diabetes Obes Metab. 2015;17(1):9–14. 25041694 10.1111/dom.12356

[54] Mann JF, Green D, Jamerson K, Ruilope LM, Kuranoff SJ, Littke T, Viberti G; ASCEND Study Group Avosentan for overt diabetic nephropathy. J Am Soc Nephrol. 2010;21(3):527–535. 20167702 10.1681/ASN.2009060593 PMC2831858

