# Supplementary material for: Insulin Inhibits Nrf2 Gene Expression via Heterogeneous Nuclear Ribonucleoprotein F/K in Diabetic Mice
Source: Endocrinology. 2017 Jan 23;158(4):903–19. doi: 10.1210/en.2016-1576 (PMC5460794; doi:10.1210/en.2016-1576)
Supplement: Supplementary file 1 [file en.2016-1576.sf1.pptx]

## Slide 1
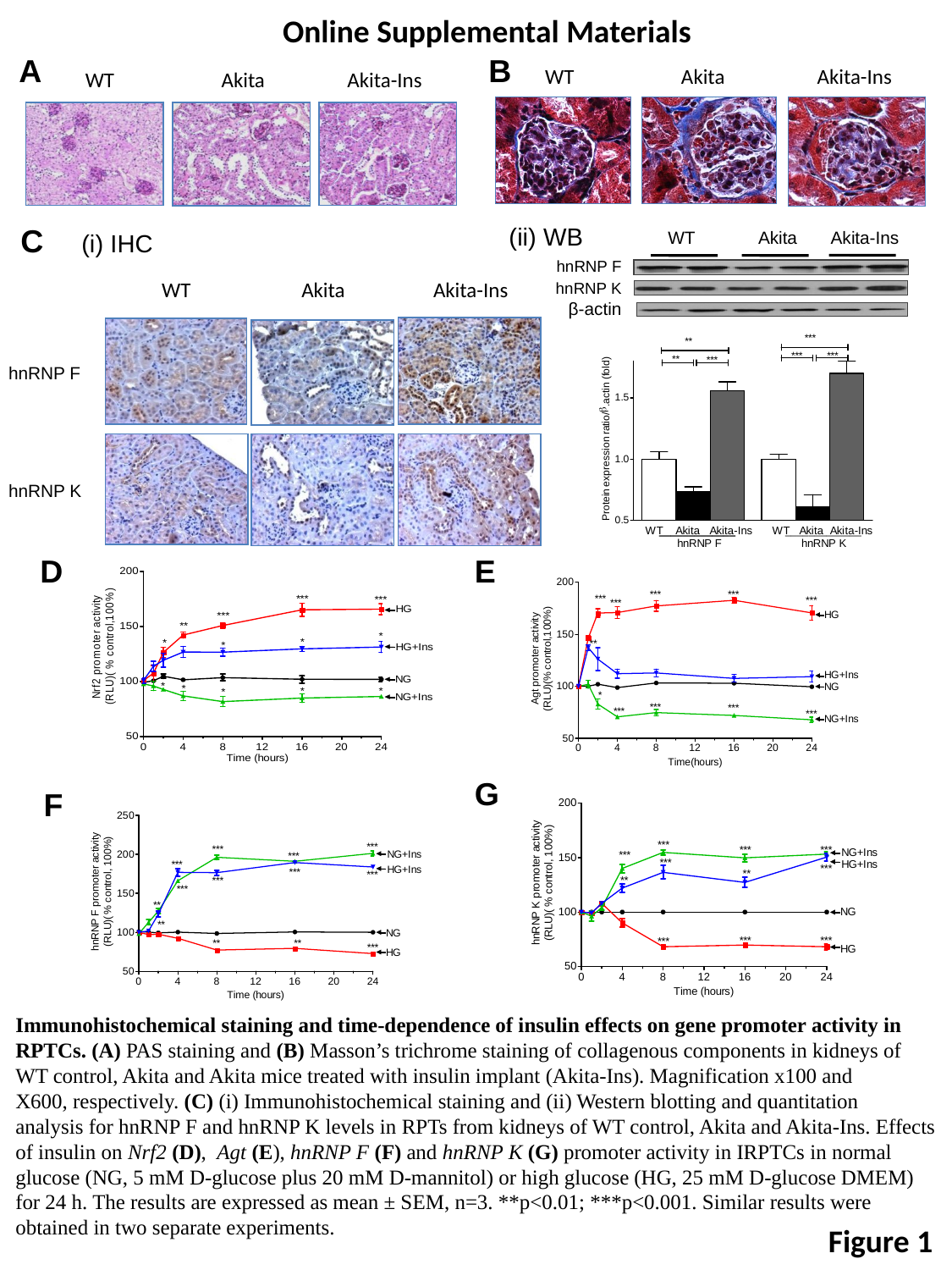

Online Supplemental Materials
A
B
WT
Akita
Akita-Ins
WT
Akita
Akita-Ins
C
(ii) WB
WT
Akita
Akita-Ins
(i) IHC
hnRNP F
WT
Akita
Akita-Ins
hnRNP K
β-actin
hnRNP F
hnRNP K
E
D
G
F
Immunohistochemical staining and time-dependence of insulin effects on gene promoter activity in RPTCs. (A) PAS staining and (B) Masson’s trichrome staining of collagenous components in kidneys of
WT control, Akita and Akita mice treated with insulin implant (Akita-Ins). Magnification x100 and
X600, respectively. (C) (i) Immunohistochemical staining and (ii) Western blotting and quantitation
analysis for hnRNP F and hnRNP K levels in RPTs from kidneys of WT control, Akita and Akita-Ins. Effects of insulin on Nrf2 (D), Agt (E), hnRNP F (F) and hnRNP K (G) promoter activity in IRPTCs in normal glucose (NG, 5 mM D-glucose plus 20 mM D-mannitol) or high glucose (HG, 25 mM D-glucose DMEM) for 24 h. The results are expressed as mean ± SEM, n=3. **p<0.01; ***p<0.001. Similar results were obtained in two separate experiments.
Figure 1

## Slide 2
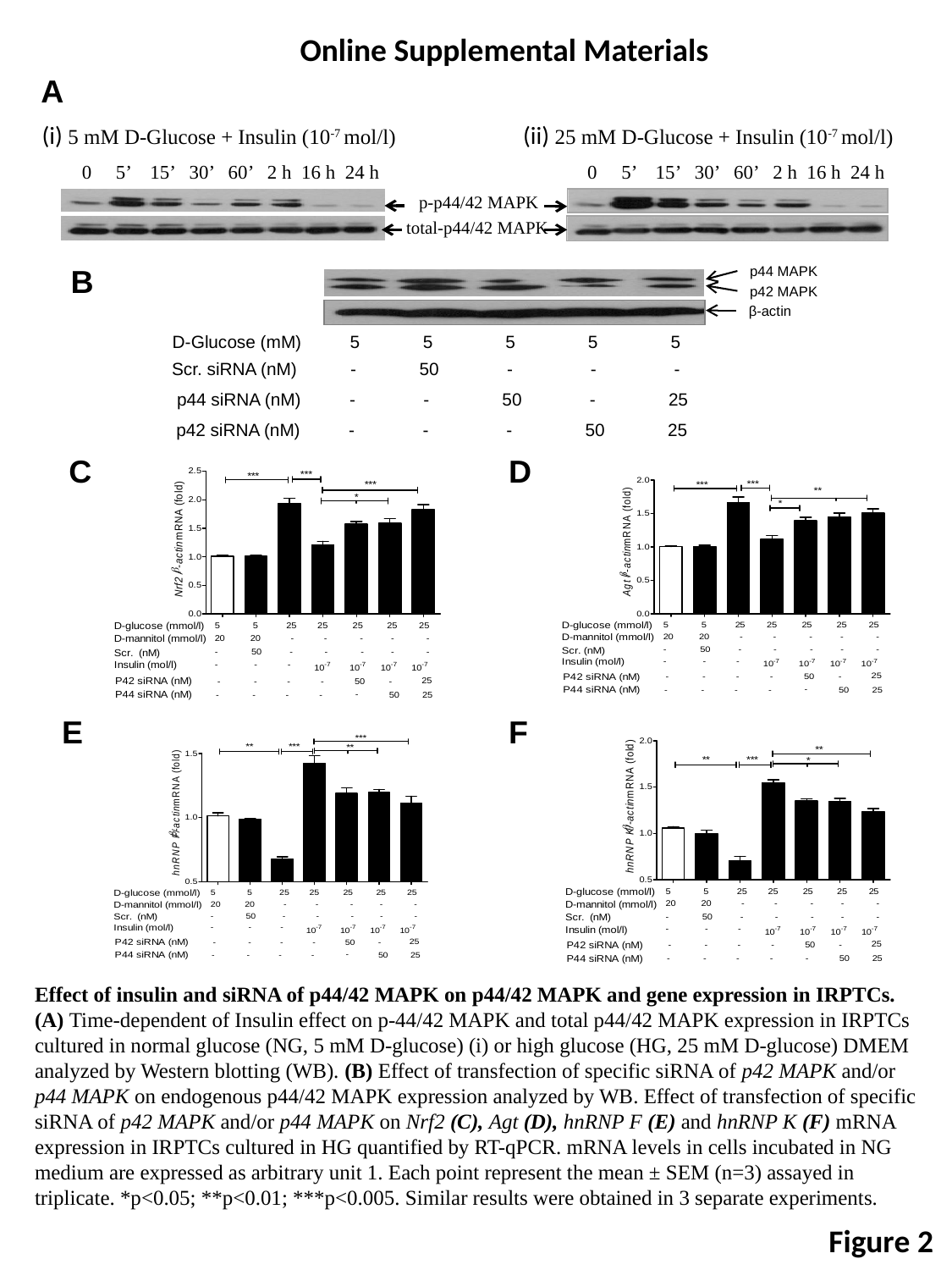

Online Supplemental Materials
A
(i) 5 mM D-Glucose + Insulin (10-7 mol/l)
(ii) 25 mM D-Glucose + Insulin (10-7 mol/l)
0 5’ 15’ 30’ 60’ 2 h 16 h 24 h
0 5’ 15’ 30’ 60’ 2 h 16 h 24 h
p-p44/42 MAPK
total-p44/42 MAPK
B
p44 MAPK
p42 MAPK
β-actin
D-Glucose (mM) 5 5 5 5 5
Scr. siRNA (nM) - 50 - - -
p44 siRNA (nM) - - 50 - 25
p42 siRNA (nM) - - - 50 25
C
D
E
F
Effect of insulin and siRNA of p44/42 MAPK on p44/42 MAPK and gene expression in IRPTCs.
(A) Time-dependent of Insulin effect on p-44/42 MAPK and total p44/42 MAPK expression in IRPTCs
cultured in normal glucose (NG, 5 mM D-glucose) (i) or high glucose (HG, 25 mM D-glucose) DMEM
analyzed by Western blotting (WB). (B) Effect of transfection of specific siRNA of p42 MAPK and/or
p44 MAPK on endogenous p44/42 MAPK expression analyzed by WB. Effect of transfection of specific
siRNA of p42 MAPK and/or p44 MAPK on Nrf2 (C), Agt (D), hnRNP F (E) and hnRNP K (F) mRNA
expression in IRPTCs cultured in HG quantified by RT-qPCR. mRNA levels in cells incubated in NG
medium are expressed as arbitrary unit 1. Each point represent the mean ± SEM (n=3) assayed in
triplicate. *p<0.05; **p<0.01; ***p<0.005. Similar results were obtained in 3 separate experiments.
Figure 2

## Slide 3
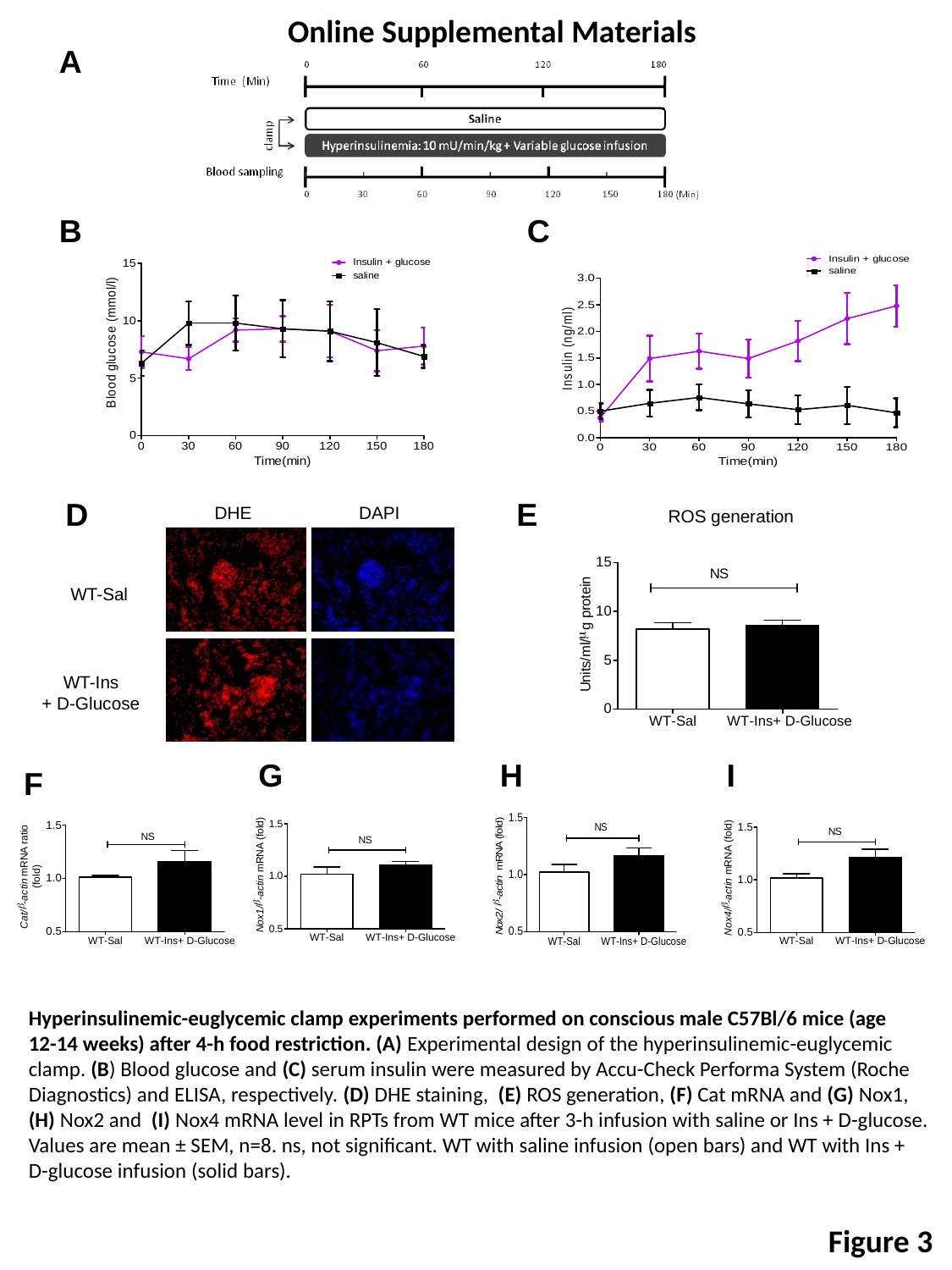

Online Supplemental Materials
A
B
C
E
D
DHE
DAPI
ROS generation
WT-Sal
WT-Ins
+ D-Glucose
G
H
I
F
Hyperinsulinemic-euglycemic clamp experiments performed on conscious male C57Bl/6 mice (age
12-14 weeks) after 4-h food restriction. (A) Experimental design of the hyperinsulinemic-euglycemic clamp. (B) Blood glucose and (C) serum insulin were measured by Accu-Check Performa System (Roche Diagnostics) and ELISA, respectively. (D) DHE staining, (E) ROS generation, (F) Cat mRNA and (G) Nox1,
(H) Nox2 and (I) Nox4 mRNA level in RPTs from WT mice after 3-h infusion with saline or Ins + D-glucose. Values are mean ± SEM, n=8. ns, not significant. WT with saline infusion (open bars) and WT with Ins +
D-glucose infusion (solid bars).
Figure 3

## Slide 4
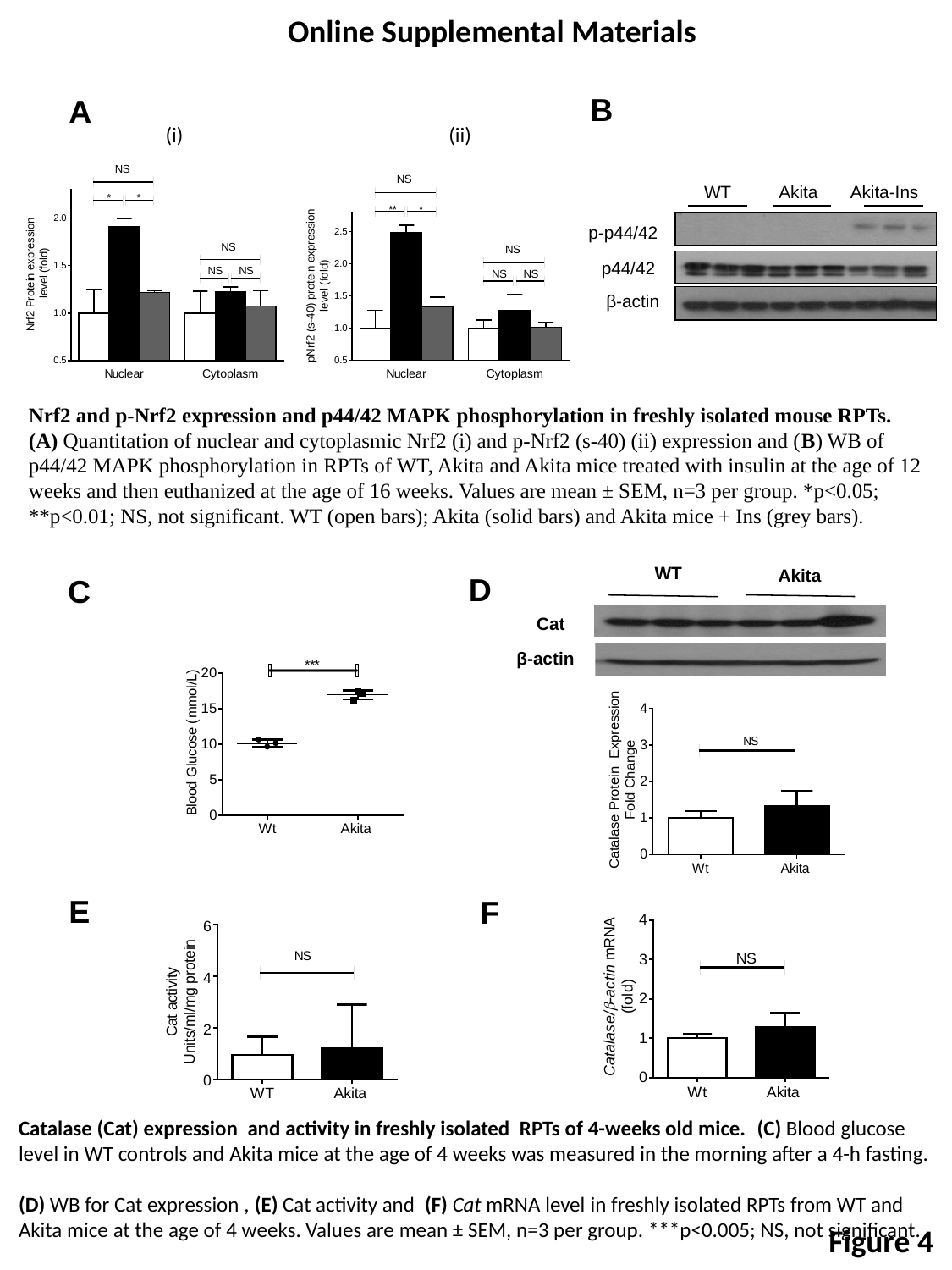

Online Supplemental Materials
B
A
(i)
(ii)
WT
Akita
Akita-Ins
p-p44/42
p44/42
β-actin
Nrf2 and p-Nrf2 expression and p44/42 MAPK phosphorylation in freshly isolated mouse RPTs.
(A) Quantitation of nuclear and cytoplasmic Nrf2 (i) and p-Nrf2 (s-40) (ii) expression and (B) WB of p44/42 MAPK phosphorylation in RPTs of WT, Akita and Akita mice treated with insulin at the age of 12 weeks and then euthanized at the age of 16 weeks. Values are mean ± SEM, n=3 per group. *p<0.05; **p<0.01; NS, not significant. WT (open bars); Akita (solid bars) and Akita mice + Ins (grey bars).
WT
Akita
D
C
Cat
β-actin
E
F
Catalase (Cat) expression and activity in freshly isolated RPTs of 4-weeks old mice. (C) Blood glucose
level in WT controls and Akita mice at the age of 4 weeks was measured in the morning after a 4-h fasting.
(D) WB for Cat expression , (E) Cat activity and (F) Cat mRNA level in freshly isolated RPTs from WT and Akita mice at the age of 4 weeks. Values are mean ± SEM, n=3 per group. ***p<0.005; NS, not significant.
Figure 4

## Slide 5
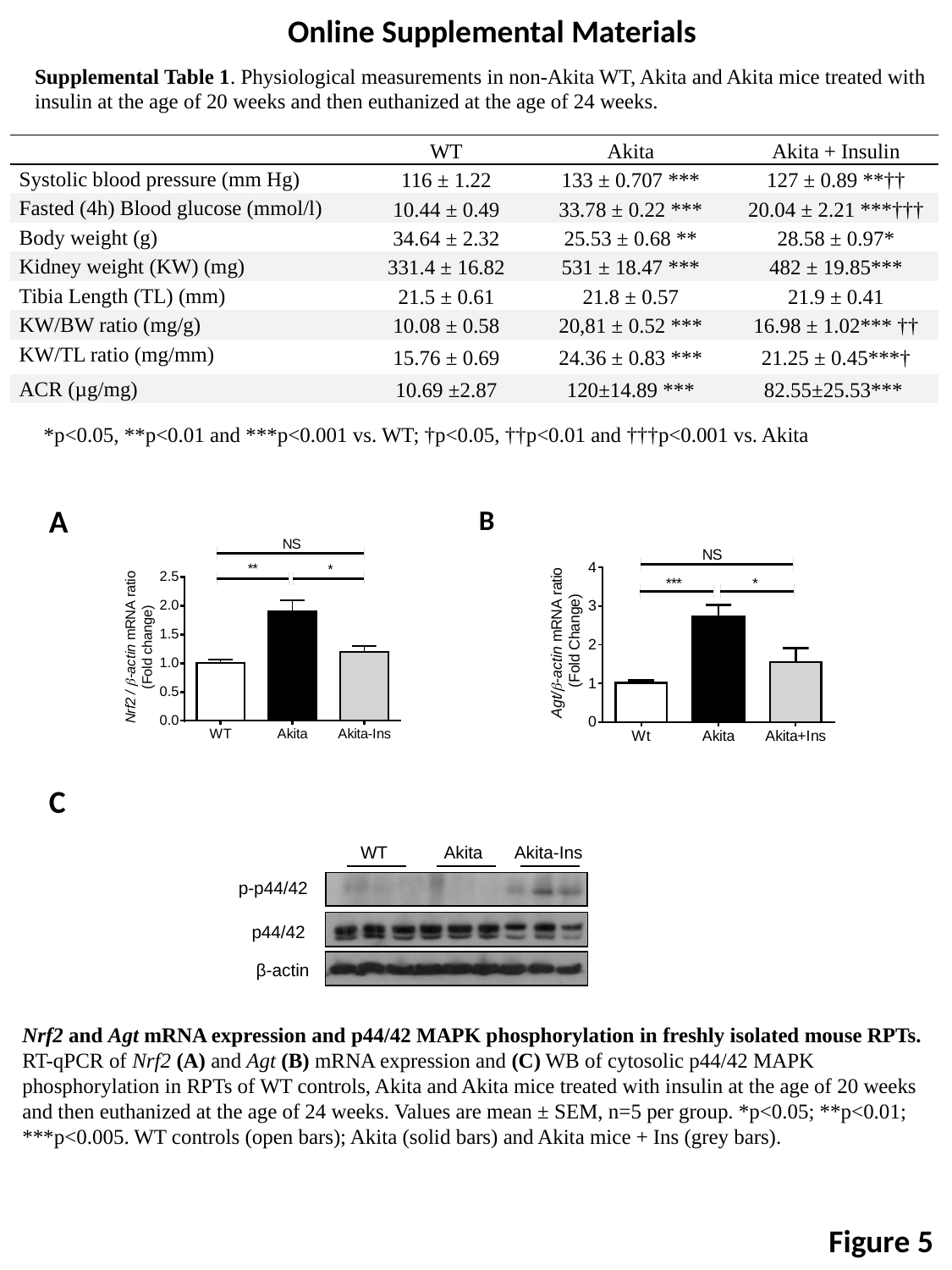

Online Supplemental Materials
Supplemental Table 1. Physiological measurements in non-Akita WT, Akita and Akita mice treated with
insulin at the age of 20 weeks and then euthanized at the age of 24 weeks.
| | WT | Akita | Akita + Insulin |
| --- | --- | --- | --- |
| Systolic blood pressure (mm Hg) | 116 ± 1.22 | 133 ± 0.707 \*\*\* | 127 ± 0.89 \*\*†† |
| Fasted (4h) Blood glucose (mmol/l) | 10.44 ± 0.49 | 33.78 ± 0.22 \*\*\* | 20.04 ± 2.21 \*\*\*††† |
| Body weight (g) | 34.64 ± 2.32 | 25.53 ± 0.68 \*\* | 28.58 ± 0.97\* |
| Kidney weight (KW) (mg) | 331.4 ± 16.82 | 531 ± 18.47 \*\*\* | 482 ± 19.85\*\*\* |
| Tibia Length (TL) (mm) | 21.5 ± 0.61 | 21.8 ± 0.57 | 21.9 ± 0.41 |
| KW/BW ratio (mg/g) | 10.08 ± 0.58 | 20,81 ± 0.52 \*\*\* | 16.98 ± 1.02\*\*\* †† |
| KW/TL ratio (mg/mm) | 15.76 ± 0.69 | 24.36 ± 0.83 \*\*\* | 21.25 ± 0.45\*\*\*† |
| ACR (µg/mg) | 10.69 ±2.87 | 120±14.89 \*\*\* | 82.55±25.53\*\*\* |
*p<0.05, **p<0.01 and ***p<0.001 vs. WT; †p<0.05, ††p<0.01 and †††p<0.001 vs. Akita
A
B
C
WT
Akita
Akita-Ins
p-p44/42
p44/42
β-actin
Nrf2 and Agt mRNA expression and p44/42 MAPK phosphorylation in freshly isolated mouse RPTs. RT-qPCR of Nrf2 (A) and Agt (B) mRNA expression and (C) WB of cytosolic p44/42 MAPK phosphorylation in RPTs of WT controls, Akita and Akita mice treated with insulin at the age of 20 weeks and then euthanized at the age of 24 weeks. Values are mean ± SEM, n=5 per group. *p<0.05; **p<0.01; ***p<0.005. WT controls (open bars); Akita (solid bars) and Akita mice + Ins (grey bars).
Figure 5

## Slide 6
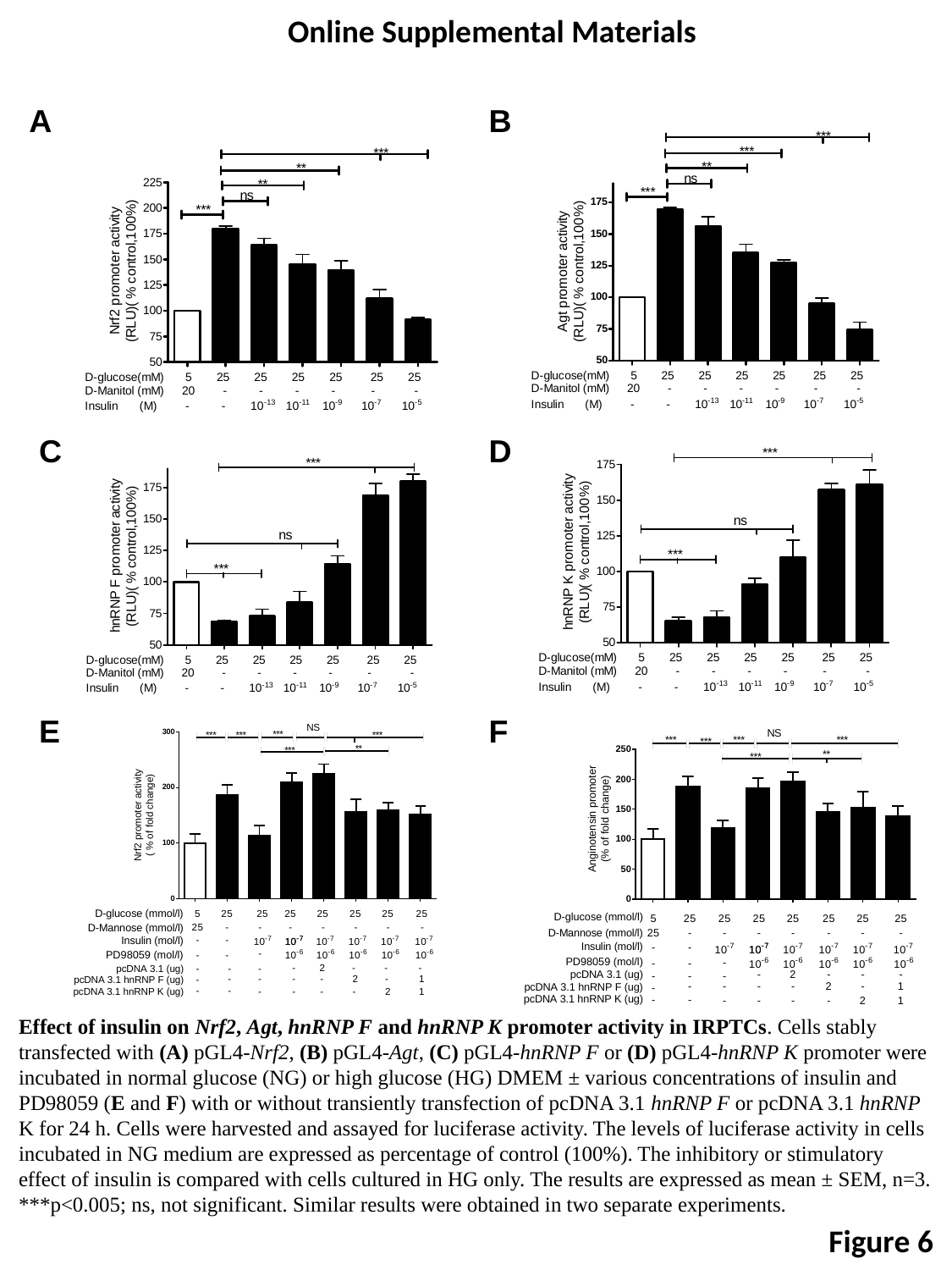

Online Supplemental Materials
A
B
C
D
E
F
Effect of insulin on Nrf2, Agt, hnRNP F and hnRNP K promoter activity in IRPTCs. Cells stably transfected with (A) pGL4-Nrf2, (B) pGL4-Agt, (C) pGL4-hnRNP F or (D) pGL4-hnRNP K promoter were incubated in normal glucose (NG) or high glucose (HG) DMEM ± various concentrations of insulin and PD98059 (E and F) with or without transiently transfection of pcDNA 3.1 hnRNP F or pcDNA 3.1 hnRNP K for 24 h. Cells were harvested and assayed for luciferase activity. The levels of luciferase activity in cells incubated in NG medium are expressed as percentage of control (100%). The inhibitory or stimulatory effect of insulin is compared with cells cultured in HG only. The results are expressed as mean ± SEM, n=3. ***p<0.005; ns, not significant. Similar results were obtained in two separate experiments.
Figure 6
